# A comparative plastomics approach reveals available molecular markers for the phylogeographic study of *Dendrobium huoshanense*, an endangered orchid with extremely small populations

**DOI:** 10.1002/ece3.6277

**Published:** 2020-04-30

**Authors:** Zhitao Niu, Zhenyu Hou, Mengting Wang, Meirong Ye, Benhou Zhang, Qingyun Xue, Wei Liu, Xiaoyu Ding

**Affiliations:** ^1^ College of Life Sciences Nanjing Normal University Nanjing China; ^2^ Jiangsu Provincial Engineering Research Center for Technical Industrialization for Dendrobium Nanjing China

**Keywords:** comparative plastomic approaches, cpSSR, *Dendrobium huoshanense*, hotspot regions, phylogeographic study

## Abstract

Comparative plastomics approaches have been used to identify available molecular markers for different taxonomic level studies of orchid species. However, the adoption of such methods has been largely limited in phylogeographic studies. Therefore, in this study, *Dendrobium huoshanense*, an endangered species with extremely small populations, was used as a model system to test whether the comparative plastomic approaches could screen available molecular markers for the phylogeographic study. We sequenced two more plastomes of *D*. *huoshanense* and compared them with our previously published one. A total of 27 mutational hotspot regions and six polymorphic cpSSRs have been screened for the phylogeographic studies of *D*. *huoshanense*. The cpDNA haplotype data revealed that the existence of haplotype distribution center was located in Dabieshan Mts. (Huoshan). The genetic diversity and phylogenetic analyses showed that the populations of *D*. *huoshanense* have been isolated and evolved independently for long period. On the contrary, based on cpSSR data, the genetic structure analysis revealed a mixed structure among the populations in Anhui and Jiangxi province, which suggested that the hybridization or introgression events have occurred among the populations of *D. huoshanense*. These results indicated that human activities have played key roles in shaping the genetic diversity and distributional patterns of *D.* *huoshanense*. According to our results, both two markers showed a high resolution for the phylogeographic studies of *D*. *huoshanense*. Therefore, we put forth that comparative plastomic approaches could revealed available molecular markers for phylogeographic study, especially for the species with extremely small populations.

## Graphical abstract

Based on the comparative plastomic approaches, a total of 27 mutational hotspot regions and six polymorphic cpSSRs have been selected to assess the genetic diversity and distributional patterns of *D. huoshanense*. According to our results, both two markers showed a high resolution for the phylogeographic studies of *D. huoshanense*.

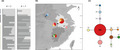

## 1 INTRODUCTION

The Orchidaceae (orchids), one of the largest families in the monocots, are well known for their tremendous diversity and innovative characters, including specialized pollination syndromes, epiphytic habits, and the presence of crassulacean acid metabolism (Chase, Cameron, Barrett, & Freudenstein, 2003; Silvera, Santiago, Cushman, & Winter, 2009). Owing to these unique features, orchids are ideal examples for phylogeographic studies, which investigating the mechanisms and processes of geographical distribution in flowering plants (Avise, 2000). However, data on such studies are still limited. Moreover, the human activities, for example, destruction or alteration of the natural environment and the overexploitation of economic plants, have led to the extinction or decline of many orchid species. For example, *Dendrobium huoshanense* C. Z. Tang & S. J. Cheng, an endangered herb endemic to China, has been extensively used as traditional Chinese medicine (TCM) in many Asian countries for hundreds of years. It is believed by TCM practitioners to contain great medical values, such as nourishing the kidney, clearing away “heat‐evil,” benefiting the stomach, enhancing the body's immunity, resisting cancer, and prolonging life. These purported medicinal merits of *D. huoshanense* bring a great market demand for it. As a perennial plant, *D*. *huoshanense* has a long‐life cycle and require a warm and humid habitat; however, due to their low germination rate, slow growing, habitat deterioration, and being overexploited, the wild populations of *D*. *huoshanense* are extremely in danger of extinction. As documented in Tsi (1999) and Wood (2006), *D*. *huoshanense* has a narrow geographical distribution, only distributed in southwest Anhui province, southwest Henan province, and a small part of Jiangxi province (Figure S1). It has been listed in the “Conservation Program for Wild Plants with Extremely Small Population in China” (State Forestry Administration of China, 2012). So, it is urgent to assess the genetic diversity and distributional patterns of *D*. *huoshanense*. However, the narrow geographical distribution and small population size of *D*. *huoshanense* have resulted in great challenges for its phylogeographic study.

The noncoding sequences in chloroplast genomes (i.e., plastomes) are the most important genetic tools for plant studies at low taxonomic levels, especially for phylogeographic analysis (Beheregaray, 2008; Prince, 2015). As reviewed in Morris and Shaw (2018), more than two‐thirds of the publications in the past 10 years employed noncoding chloroplast regions alone or in combination with nuclear DNA sequences or microsatellites. For instance, in *Dendrobium* orchids, Ye et al. (2016) evaluated the ecological and genetic processes of *D. moniliforme* by using two noncoding chloroplast (cp) DNA sequence (*trnC*‐*petN* and *trnT*‐*trnE*). Hou et al. (2017) elucidated the iteration expansion and regional evolution history of *D. officinale* and four related taxa by employing the sequence combination of nuclear ITS regions and three cpDNA regions (*accD*‐*psaI*, *trnC*‐*petN*, and *rps15*‐*ycf1*). Unfortunately, though cpDNA sequence data use increase, most researchers continue rely on regions of relatively low variability. For example, the commonly used cpDNA regions, *psbA*‐*trnH*, *trnL*‐*trnF,* and *trnL* intron present lower variability than most of other regions (Morris & Shaw, 2018). Besides, most of cpDNA sequences do not provide sufficient resolution for the phylogeographic studies of plant species with extremely small populations.

Advances in next‐generation sequencing (NGS) approach over the last decade has largely facilitated gathering of plastome sequence data in public databases, which led to the use of comparative plastomics approaches (i.e., comparisons of sequences variability among different regions at whole‐plastomic level) to identify rapidly evolving regions appropriate for low taxonomic level studies use (e.g., Ahmed et al., 2013; Niu, Xue, et al., 2017; Shaw et al., 2014). Recently, numerous mutational hotspot regions have been screened in different orchid genera, for example, *Cymbidium* (Yang, Tang, Li, Zhang, & Li, 2013), *Phalaenopsis* (Shaw et al., 2014), *Holcoglossum* (Li et al., 2019) and especially in the genus of *Dendrobium*. For instance, the five informative hotspot regions *trnT*‐*trnL*, *rpl32*‐*trnL*, *clpP*‐*psbB*, *trnL* intron, and *rps16*‐*trnQ* were selected for identifying species of *Dendrobium* (Niu, Zhu, et al., 2017). On the basis of comprehensive plastome‐wide comparison, a total of 19 SNPs were used to design RT‐ARMS primers for the authentication study of *Dendrobium* species (Niu et al., 2018). While the potential benefit of comparative plastomics approaches to low taxonomic level study has become increasingly clear, the adoption of such methods has been largely limited in phylogeographic studies. At least for now, plant phylogeographic studies in *Dendrobium* orchids remain dependent on the noncoding cpDNA sequences (e.g., Hou et al., 2017; Ye et al., 2016).

Therefore, in this study, the plastome sequences of *D*. *huoshanense* were used as a model system to address two questions, as follows: (a) Could the comparative plastomic approaches screen available molecular markers? (b) If so, could these markers use to infer the phylogeographic relationships among the populations of *D*. *huoshanense*. To address these questions, we sequenced two more complete plastome sequences of *D*. *huoshanense* and compared them with our previously published one. Based on the comparative plastomic approaches, a total of 27 mutational hotspot regions and six polymorphic cpSSRs have been selected to assess the genetic diversity and distributional patterns of *D. huoshanense*.

## 2 MATERIALS AND METHODS

### 2.1 Plant materials and DNA extraction

A total of 28 samples from five populations of *D. huoshanense* were used in this study (Table 1). These plant samples were collected from 2015 to 2018 in Anhui, Guangxi and Henan provinces of China. We only harvested leaves of *D. huoshanense* nondestructive and left the vulnerable wild population intact. In addition, 6 samples of *D. moniliforme* from Anhui (voucher numbers: Niu18071‐Niu18075, Niu19022) province were collected as outgroups. The voucher specimens of *D. huoshanense* (Niu16009, Niu20003‐Niu20006) and all samples were stored in College of Life Sciences, Nanjing Normal University, Nanjing, China.

**TABLE 1:** Sampling locations and population sizes of five *D. huoshanense* populations

Population	Population location	Altitude (m)	Latitude (*N*°)	Longitude (E°)	Number of individuals sampled from this population
Dabieshan Mts. (Huoshan)	Huoshan, Anhui	1,011	31.029	116.053	9
Dabieshan Mts. (Lu'an)	Lu'an, Hubei	1,064	31.101	115.793	7
Huangshan Mts.	Huangshan, Anhui	889	30.175	118.183	2
Longhushan Mts.	Longhushan, Jiangxi	925	26.555	114.156	6
Funiushan Mts.	Nanzhao, Henan	986	33.633	111.552	4

Note

“Mountains” were abbreviated to “Mts.”.

For the DNA extraction of two individuals from Dabieshan Mts. (Huoshan) and Longhushan Mts., 2 g of fresh leaves were harvested. The total genomic DNA was extracted using the DNeasy Plant Mini Kit (Qiagen) according to the manufacturer's instructions. The quality of obtained DNA was measured on NanoDrop 8,000 Spectrophotometer (Thermo Scientific). DNA samples that met the quality requirements (concentration >50 ng/μl, A260/A280 = 1.8–2.0, and A260/A230 >1.8) were used for sequencing. The genomic DNA of other samples were isolated from about 0.5 g of leaves using a standard CTAB DNA extraction protocol (Doyle & Doyle, 1987) modified by adding 2% PVP‐40 to the buffer.

### 2.2 Plastome sequencing, assembly, and annotation

The total genomic DNA of two individuals was sequenced using an Illumina Hiseq4000 sequencer with the pair‐end strategy of 150 bp reads with an average 400 bp insert size. Approximately, 5.5 Gb raw reads were yielded for each species. The raw reads were trimmed with an error probability < .05 and by removing one nucleotide at both terminal ends, and then assembled on CLC Genomics Workbench 8.5.1 (CLC Bio) with the plastome of *D*. *moniliforme* (AB893950) as reference. The gaps and junctions between inverted repeat (IR) regions and single copy (SC) regions were confirmed by PCR amplification. Genes were annotated using DOGMA (Wyman, Jansen, & Boore, 2004) and tRNAscan‐SE 1.21 (Schattner, Brooks, & Lowe, 2005). The exact boundaries of annotated genes were manually checked by aligning them with homologous genes of other plastomes in the genus of *Dendrobium*.

### 2.3 Comparative plastomics analyses

The comparative plastomics approaches were employed to select the most informative regions, which could be used for the phylogeographic analysis of *D. huoshanense*. Three plastomes of *D. huoshanense* were compared by using *D*. *moniliforme* (AB893950) as reference. The complete plastome sequences were aligned with MAFFT program (Katoh & Standley, 2013) and adjusted manually in MEGA 5.2 (Tamura, 2011). The ambiguous sites were removed. Then, we divide the aligned sequences into 372 bins with 400 bp per length using *D*. *moniliforme* as outgroup. DnaSP v5 was employed to count the numbers of single nucleotide polymorphism (SNP) sites and insertion‐deletion (InDel) events for each bin (Librado & Rozas, 2009). Chloroplast simple sequence repeat (cpSSR) elements were also detected using GMATo according to the criteria that the “Mini‐length” for mono‐nucleotide and multi‐nucleotide SSRs were set to be 8 and 5 units, respectively (Wang, Lu, & Luo, 2013). Correlation analyses were performed by using SPSS Statistics 20.0. Plastome map was drawn by using Circos v0.67 (http://circos.ca/) and manually refined with Adobe Illustrator CC 2015.

### 2.4 Population genetic analyses

A total of 27 mutational hotspot regions were sequenced from 28 samples of five populations of *D*. *huoshanense* (Table S1). The amplified sequences were aligned using MUSCLE (Edgar, 2004) with the “Refining” option implemented in MEGA 5.2. Gaps located at the 5′‐ and 3′‐ends of alignments and all mononucleotide repeats were excluded. Indels were treated as single mutation events. We used DnaSP v5 to identify haplotypes, and calculated the haplotype diversity (*H_d_*) and nucleotide diversity (*P_i_*) for each population and for all samples. Within‐population diversity (*H_S_*) and total diversity (*H_T_*) were determined using PERMUT version 1.0 (Pons & Petit, 1996). For the analyses of molecular variance (AMOVA): Firstly, we divided the five population into three groups, (a) Dabieshan Mts. (Huoshan), Dabieshan Mts. (Lu'an), Huangshan Mts. (Anhui province), (b) Longhushan Mts. (Jiangxi province), (c) Funiushan Mts. (Henan province). Then, AMOVA analyses were performed to quantify the genetic differentiation within populations, between populations within groups, and between groups using ARLEQUIN version 3.5, with 1,000 random permutations (Excofer & Lischer, 2010). To detect the presence of phylogeographic structure, the difference between *G_ST_* and *N_ST_* was estimated with PERMUT version 1.0. Unique haplotype sequences were submitted to DNA Data Bank of Japan to obtain accession numbers.

### 2.5 Phylogeographic analyses

Phylogenetic analyses of the 11 haplotypes were assessed via Bayesian inference (BI) analyses. The best substitution model was “GTR + I+ G”, which was determined according to the Akaike Information Criterion (AIC) in Modeltest 3.7 (Posada, 2008). The BI tree was constructed using MrBayes 3.2 (Ronquist et al., 2012). Two simultaneous runs were conducted, each consisting of four chains. In total, chains were run for 2,000,000 generations, with topologies sampled every 100 generations. The first 25% of our sampled trees were discarded. The remaining trees were used to construct a majority‐rule consensus tree and calculate posterior probabilities (PPs) for each node. In addition, we used NETWORK version 4.5 to build a maximum‐parsimony median‐joining network to visualize the phylogenetic relationships among all the 11 haplotypes (Bandelt, Forster, & Rohl, 1999).

### 2.6 Structure analysis

The model‐based program STRUCTURE was used to determine the genetic structure of five populations of *D*. *huoshanense* by using cpSSR data (Falush, Stephens, & Pritchard, 2003). We used a burn‐in of 500,000 and MCMC iteration number = 1,200,000, assumed an admixture model and correlated allele frequencies, and included no prior information on taxon identity; default values were used for all other parameters. The number of groups (*K*) was varied from 1 to 7 with 10 independent runs. The most probable number of clusters was estimated by calculating the natural logarithm of the likelihood function, and the Δ*K* statistic was estimated using Structure Harvester (Earl, Vonholdt, Earl, & VonHoldt, 2012).

## 3 RESULTS

### 3.1 Genome features

The two newly sequenced plastomes of *D*. *huoshanense* were 148,786 and 149,186 bp in size, which were similar to the already reported plastome sizes of related *Dendrobium* species (e.g., Niu, Zhu, et al., 2017). The plastomes were circular and possessed typical quadripartite structure, which included a pair of inverted repeats (i.e., IR_A_ and IR_B_) (25,984 and 25,966 bp) and separated SSC (12,099 and 12,087 bp) and LSC (84,719 and 85,167 bp) regions (Figure S2). The overall AT content were 37.53 and 37.66%, respectively, indicating nearly identical levels among the *Dendrobium* plastomes. Moreover, the overall genomic structure including gene number and gene order were also well‐conserved. A total of 103 functional genes were encoded in the plastome of *D*. *huoshanense*, consisting of 69 unique protein‐coding genes, 30 unique tRNA genes, and four unique rRNA genes. The sequence of eleven plastid NDH genes was compared with *Apostasia odorata* (NC_030722) which contains full set of functional NDH genes in orchids. Like other *Dendrobium* species, *D*. *huoshanense* also experienced the loss of plastid NDH genes. Among them, only *ndhB* genes in IR regions contain full reading frames, whereas other ten plastid NDH genes were truncated or completely lost.

### 3.2 Comparative plastomics analyses of *D*. *huoshanense*

**FIGURE 1:**
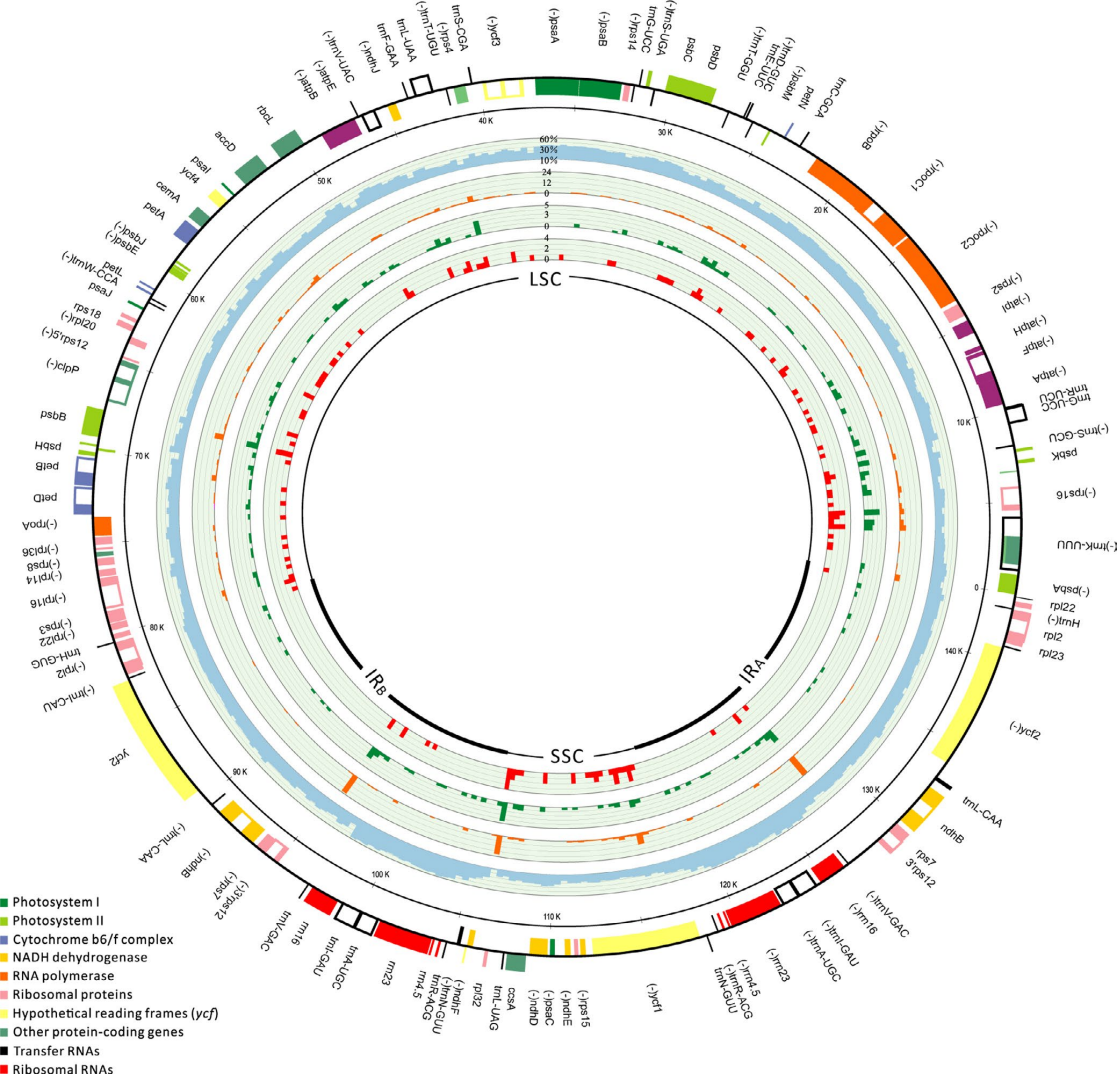
Comparative plastomic analyses of *D*. *huoshanense*. Plastome sequences were aligned and divided into 372 bins with 400 bp per length by using *D*. *moniliforme* (AB893950) as reference. Color boxes from the outermost to innermost indicate (1) CG content, (2) number of SNPs, (3) number of InDel events, and (4) number of cpSSRs.

A total of 372 bins with 400 bp per length were achieved from three plastomes. The relationship between GC content and distribution of SNP and InDel in pairwise comparison of each bins were showed in Figure 1 and Table S2. The correlation analyses showed that the distribution of SNP was correlated with InDel (Spearman's *r* = .406, *p* < .01). Moreover, negative correlations between GC content and the distribution of SNP and InDel were also detected (Spearman's, *r* = −.473, −.267, all *p* < .01). These results indicated that the plastomic mutational hotspots of *D*. *huoshanense* were accompanied by biased AT compositions. In addition, we also calculated the number of SSRs that present in the same loci of both plastomes (Figure 1). It has been reported that the SSRs were predisposed to distribute in mutational hotspot regions (Ahmed et al., 2013). Consistently, our study showed that the distribution of SSR was correlated with SNP and InDel (*r* = .365, .358, all *p* < .01).

According to comparison results, two classes of molecular markers, plastomic sequences and polymorphic cpSSRs, have been selected. A total of 27 regions, with the highest number of SNPs and InDels, were screened as the mutational hotspot regions, which can be used to infer the phylogeographic relationships among five populations of *D*. *huoshanense* (Table S3). Besides, six polymorphic cpSSRs were identified in those regions, which could be used to evaluate genetic structure of each *D. huoshanense* populations (Table S4).

### 3.3 Sequence variation and genetic diversity

These 27 mutational hotspot regions were sequenced from 28 samples of five populations of *D*. *huoshanense*, with aligned length of 10,415 bp. Based on sequence variation analysis, a total of 11 haplotypes were identified, which contains 14 polymorphic sites, including 9 SNPs and 5 InDel mutations (Table S5, LC494015–LC494038). Genetic analysis revealed that the total genetic diversity (*H_t_* = 0.942) of all sampled populations was substantially higher than the average within‐population diversity (*H_s_* = 0.663). The high levels of haplotype diversity (*H_d_* = 0.815) and nucleotide diversity (*P_i_* = 0.00017) were estimated at species level, but diversified at population level. The highest level of haplotype diversity was 0.810 in the population of Dabieshan Mts. (Lu'an), followed by Dabieshan Mts. (Huoshan) and Longhushan Mts., while, the value of haplotype diversity was estimated 0 in both Huangshan Mts. and Funiushan Mts. (Table 2).

**TABLE 2:** Haplotype diversity, nucleotide diversity, and haplotype frequency in each population

Population	Number of haplotypes	*H_d_*	*P_i_*	Haplotype (number of samples)
Dabieshan Mts. (Huoshan)	5	0.750	0.00019	H1(2), H2(4), H3(1), H4(1), H5(1)
Dabieshan Mts. (Lu'an)	4	0.810	0.00012	H2(3), H4(1), H5(1), H6(2)
Huangshan Mts.	1	0.000	0.00000	H6(2)
Longhushan Mts.	4	0.733	0.00013	H1(1), H2(2), H7(2), H8(1)
Funiushan Mts.	3	0.000	0.00000	H9(2), H10(1), H1(1)
All groups	11	0.815	0.00017	

Abbreviations: *H_d_*, haplotype diversity; *P_i_*, nucleotide diversity.

The AMOVA analysis showed that: (a) The genetic variation within populations (88.39%) was significantly higher than that among populations (11.61%). The estimated value of genetic differentiation (*Φ_ST_*) was 0.1161. (ii) Among three groups, the total genetic variation was partitioned into 9.10% among groups, 4.85% among populations within groups, and 86.05% within populations (Table 3). The values of genetic differentiation parameters *Φ_CT_*, *Φ_SC_*, *Φ_ST_* were 0.0910, 0.0533, 0.1395, respectively. Those results indicated that the genetic variation of *D*. *huoshanense* mainly occurred within populations. In addition, the estimated value of *N_ST_* (0.478) was substantially higher than *G_ST_* (0.296), revealing strong phylogeographic structure in this species.

**TABLE 3:** AMOVA results for five *D. huoshanense* populations based on cpDNA haplotype frequencies

Source of variation	*df*	Sum of squares	Variance components	Percentage of variation	Fixation indices
All populations					
Among populations	4	2.683	0.05177Va	11.61	*Φ_ST_* = 0.1161
Within populations	23	9.067	0.39424Vb	88.39	
Total	27	11.750	0.44600		
Three groups					
Among groups	2	1.613	0.04168Va	9.10	*Φ_CT_* = 0.0910
Among populations within groups	2	1.070	0.02221Vb	4.85	*Φ_SC_* = 0.0533
Within populations	23	9.067	0.39424Vc	86.05	*Φ_ST_* = 0.1395
Total	27	11.750	0.45813		

Abbreviations: *df*, degrees of freedom; *Φ_CT_*, genetic variation among groups; *Φ_SC_*, genetic variation among populations within groups; *Φ_ST_*, genetic variation within populations.

### 3.4 Phylogenetic analysis

**FIGURE 2:**
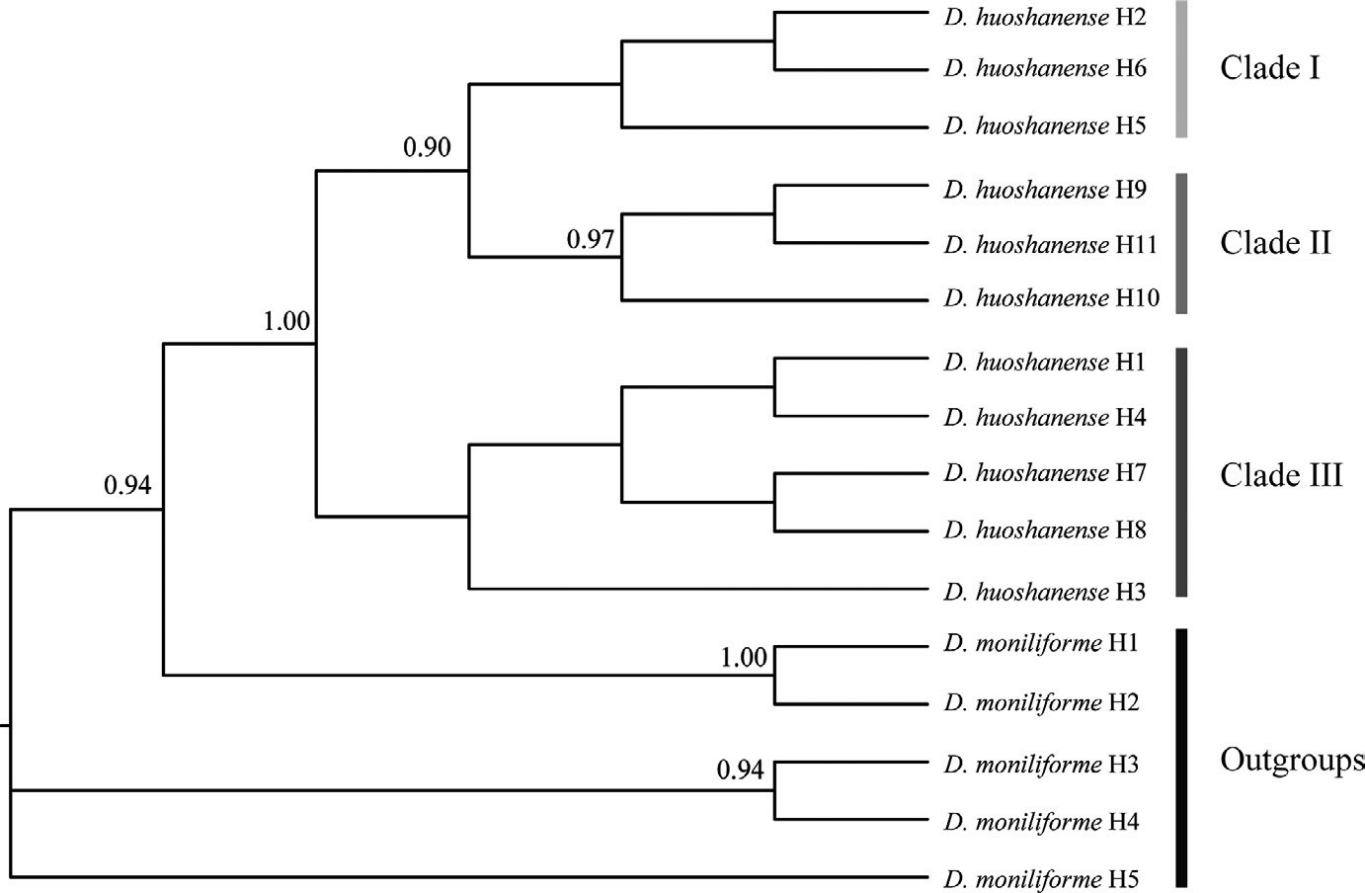
Phylogenetic relationships among the 11 haplotypes of *D*. *huoshanense*. Bayesian inference (BI) analyses were used to infer the phylogenetic relationships among *D*. *huoshanense* haplotypes with *D*. *moniliforme* as outgroup. Numbers on each node represents the posterior probability of the clade (only values > 0.5 are shown).

We performed the Bayesian inference (BI) analyses to infer the phylogenetic relationships among 11 *D*. *huoshanense* haplotypes with *D. moniliforme* as outgroup. Although it was unable to achieve high support values for all nodes, the BI tree well‐separated haplotypes into three clades: Clade I contained three haplotypes of H2, H5, H6, Clade II contained three haplotypes of H9, H10, H11, and Clades III contained five haplotypes of H1, H3, H4, H7, H8 (Figure 2, Figure S3).

**FIGURE 3:**
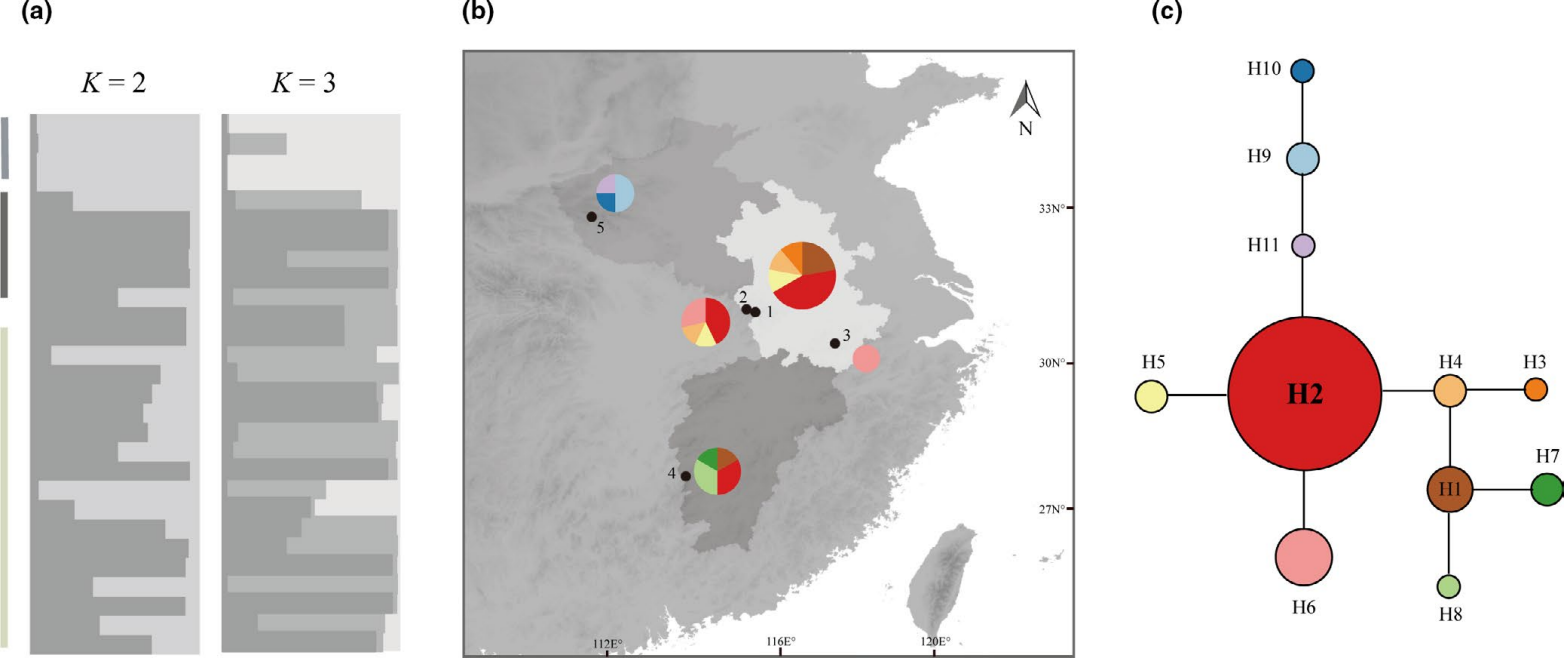
Phylogenetic analyses of *D*. *huoshanense*. (a) Genetic structure of five *D*. *huoshanense* populations for *K* = 2 and *K* = 3 chain. Populations located in three provinces were color coded: Jiangxi province = dark gray, Henan province = gray, Anhui province = light gray. (b) Map of sampling locations and geographic distribution of cpDNA haplotypes. Population locations: 1, Dabieshan Mts. (Huoshan); 2, Dabieshan Mts. (Lu'an); 3, Huangshan Mts.; 4, Longhushan Mts.; 5, Funiushan Mts.. Colors in the haplotype pies for the five populations correspond to the haplotypes in the network where size of the circle corresponds to the number of samples with the haplotype. The map was drawn using ArcGIS 10.2 (ESRI, CA, USA) and Adobe Illustrator CC 2015. (c) Network analysis of genealogical relationships among the 11 cpDNA haplotypes. The size of the circles corresponds to the frequency of each haplotype

The median‐joining analysis revealed that the network of 11 *D*. *huoshanense* haplotypes was congruent with the phylogenetic tree, with all three clades recovered (Figure 3c). Most of the haplotypes in adjacent populations were clustered together, suggesting that geography might have played a role in shaping the extant genetic structure of *D*. *huoshanense* populations. Among them, haplotype H2 presented the highest frequency of 32.14% and geographically wide distribution, which suggested its nature of an ancestral haplotype. Notably, the population of Dabieshan Mts. (Huoshan) not only contained the highest frequency of H2 (44.45%) but also had the highest number of haplotypes, indicating that the existence of haplotype distribution centers was located in Dabieshan Mts. (Huoshan) (Figure 3b). As showed in Figure 3c, the haplotype H7 and H8 evolved from H2 by three mutational steps across H4 and H1 separately. However, the population of Longhushan Mts. had a high frequency level of H2, H7, and H8 haplotype, but lacked the haplotype H4 and H1. Moreover, haplotype H2 was absent in populations of Funiushan Mts. and Huangshan Mts., while haplotype H9, H10, and H 11 were only found in Funiushan Mts. (Figure 3b). These results implying that the three populations might have been isolated and evolved independently for long period.

### 3.5 Genetic structure analysis

A total of six polymorphic cpSSRs were employed for the genetic structure analysis. The genetic structure of each *D. huoshanense* populations was determined by means of the STRUCTURE program with the admixture model. According to estimated values of Δ*K*, when *K* = 3, the genetic structure analysis should achieve the best result (Figure S4). However, as shown in Figure 3a, the analysis revealed the similar genetic structure when *K* = 2 and 3. Based on the results, five *D. huoshanense* populations could be divided into two clusters. The population of Funiushan Mts. clustered as a relative separate cluster, while the other four populations were mixed with each other. These results indicated that the hybridization or introgression events have occurred among the populations of *D. huoshanense*.

## 4 DISCUSSION

### 4.1 Comparative plastomic approaches could revealed available molecular markers for the phylogeographic study of endangered species with extremely small populations

The term “extremely small populations” refers to a population having two essential factors: (a) with a narrow geographical distribution which has resulted from some negative external factors over a long time; (b) contains less individuals than the minimal number that required to prevent extinction (Chen et al., 2014). Indeed, most of the endangered species with extremely small populations in China are qualified as the biological resources important to the country's ecology, science, culture, and economics (Volis, 2016; Yang, Xiang, Zhang, Kang, & Shi, 2015). However, the two essential factors for species with extremely small populations have raised great challenges for the phylogeographic studies, especially for the molecular marker selection. For example, *D*. *huoshanense*, a rare and endemic herb to China, has been extensively used as traditional Chinese medicine (TCM) for hundreds of years (Bao, Shun, & Chen, 2001). Because of its excellent medicinal merits, *D*. *huoshanense* has attracted intense attention of researchers, leading to numerous studies published, which focus on biochemical (Li et al., 2018; Liang et al., 2019), pharmacological (Ge et al., 2018; Xie et al., 2019), and authentication studies (Niu et al., 2018). Moreover, to protect the endangered species and meet the great market demand, plant breeders have also raised the artificial cultivation size of *D*. *huoshanense*. However, until now, the phylogeographic relationship among *D*. *huoshanense* populations still remains unclearly due to the lack of available molecular markers.

In order to identify the most informative markers for orchid species, comparative plastomic approaches have been already used to assess sequence variabilities at different level studies, for instance, Niu, Xue, et al. (2017), Niu, Zhu, et al. (2017) compared the sequence variabilities of 68 syntenic noncoding loci among 11 orchid genera across five subfamilies; Shaw et al. (2014) calculated the sequence variabilities of 107 noncoding loci in *Cymbidium* and *Phalaenopsis* genus; Li et al. (2019) surveyed intraspecific variation in intergenic regions of *Holcoglossum* plastomes. The comparison studies have led to a better understanding of the sequence variation among orchid plastomes, but failed to screened the most informative markers, which could be used at all level studies of orchid species. The diversified mutational hotspots among different orchid genera and different species indicated that comparative plastomic approaches are required for studies of orchid species at different levels.

In this study, on the basis of comprehensive plastome‐wide comparison, two classes of molecular markers, (i) 27 mutational hotspot regions and (ii) six polymorphic cpSSRs have been selected for the phylogeographic study of *D*. *huoshanense*. Among them, 19 hotspots were located in the four regions, *ccsA* to *ndhF*, *matK* to *3′trnG*, *rpoB* to *psbD,* and *trnT* to *rbcL*, which were identified as the most informative hotspot regions in *Dendrobium* plastomes (Niu et al., 2018). Moreover, both molecular markers showed high resolution for the phylogeographic studies of *D*. *huoshanense*. These results indicated that comparative plastomic approaches could revealed available molecular markers for the phylogeographic study of endangered species with extremely small populations.

### 4.2 Human activities have played key roles in shaping the genetic diversity and distributional patterns of *D*.* huoshanense*

In the current research, a total of 11 haplotypes were identified among 28 samples of five natural populations of *D*. *huoshanense*. With carefully calculation and statistical analysis, two groups of opposite results have been revealed. (a) We have observed a high level of total genetic diversity (*H_t_* = 0.815), which implicated that *D*. *huoshanense* has a long evolutionary history and wide distribution. While the documented distribution of *D*. *huoshanense* was limited in Tsi (1999). Moreover, the haplotype diversity (*H_d_*) was diversified for different populations, for example, the values of *H_d_* were 0.810, 0.750, and 0.733 for Dabieshan Mts. (Lu'an), Dabieshan Mts. (Huoshan), and Longhushan Mts., but 0 for Huangshan Mts. and Funiushan Mts.. (b) The estimated value of *D*. *huoshanense* population differentiation based on cpDNA data (*Φ_ST_* = 0.1161) was lower than other seed plants in China (e.g., Qiu, Fu, & Comes, 2011; Wang et al., 2009; Xu et al., 2010), which is in line with a low level of genetic differentiation in other *Dendrobium* orchids (e.g., Hou et al., 2017; Ye et al., 2016). These opposite results might be caused by two factors: the unique characters of orchid species and the influence of human activities. Generally, dust‐like orchid seeds have the capacity to travel long distance through typhoons or tornadoes, promoting gene flow among populations (Adams, 2011; Li et al., 2008). However, due to the lack of endosperm and the requirement of mycorrhizal fungi for germination, orchid seeds falling close to their parental plants may have more chance to germinate (Chung & Chung, 2008; Fay, Pailler, & Dixon, 2015). Actually, only 5% seeds can be successfully germinated in the wild, contributing to limited gene flow (Phillips, Dixon, & Peakall, 2012; Swarts & Dixon, 2009). *Dendrobium* orchids commonly inhabit warm and moist habitat, being highly susceptible to environmental deterioration (Pinheiro, Cafasso, Cozzolino, & Scopece, 2015). However, Human activities, for example, destruction or alteration of the natural environment and the overexploitation have accelerated the decline of gene flow and genetic differentiation among *D*. *huoshanense* species.

Human activities not only influence the genetic diversity and structure of *D*. *huoshanense*, but also affect the distributional patterns of *D*. *huoshanense*. The network of the 11 identified cpDNA haplotypes revealed that the existence of haplotype distribution center was located in Dabieshan Mts. (Huoshan). Inferred from the BI tree, three major clades were identified in relation to geographical distribution, suggesting that geography might have played a role in shaping the extant genetic structure of *D*. *huoshanense* populations. Although most of the haplotypes were distributed in nearly all regions, there were still five haplotypes restricted to Longhushan and Funiushan Mts., indicating that these two populations were isolated for long periods (Guo, Luo, Liu, & Wang, 2015; Twyford, Kidner, & Ennos, 2015). However, based on the structure analysis, except for the population of Funiushan Mts., the populations of Dabieshan Mts. (Huoshan and Lu'an), Longhushan Mts., and Huangshan Mts. were mixed with each other indicate those regions were underwent hybridization or introgression events (Cheng, Hwang, & Lin, 2005; Hou et al., 2017). Since the early twentieth century, plant breeders in China have raised the artificial cultivation size of *D. huoshanense*, especially in Anhui and Jiangxi provinces, to protect the endangered species and meet the great market demand. They have collected *D. huoshanense* across the Dabieshan Mts. (Huoshan), and reproduced them in artificial conditions. The *D. huoshanense* species have not only been grown in greenhouses, but have also been cultivated in conditions imitating those in the wild. For example, breeders have grown *D. huoshanense* species on the trunk of pear trees or on cliffs, which has afforded an opportunity for its artificial migration. Therefore, the hybridization or introgression events would occur between cultivated and natural populations, which has resulted in the mixed genetic structure among *D*. *huoshanense* populations. Hence, human activities should have played a key role in shaping the distributional patterns of *D*. *huoshanense* over the past 100 years, but the level of artificial migration should be evaluated in the future.

In summary, based on phylogeographic analysis, we proposed that human activities have played key roles in shaping the genetic diversity and distributional patterns of *D*. *huoshanense*. To protect the wild population of *D*. *huoshanense* and meet the great market demand, the conservation strategy should be implemented in three steps: (a) establish a germplasm bank by collecting seeds from different populations; (b) raise the cultivation size and increase the gene flow among populations by artificial hybridization; (c) plant the species of *D*. *huoshanense* back into the wild.

### 4.3 Implications for phylogeographic study

The cpDNA sequences have been widely used in plant phylogeographic studies. However, with extensive comparative analyses of noncoding cpDNA sequences, Shaw et al. (2014) concluded that the most variable regions were diverse among different plant lineages. This conclusion was confirmed by our previous plastomics studies in orchid species (Niu et al., 2018; Niu, Xue, et al., 2017; Niu, Zhu, et al., 2017), which indicated that marker screening is necessary before subsequent phylogeographic or other low taxonomic studies. Nevertheless, as reviewed in Morris and Shaw (2018), it is common for authors to indicated that they had screened several markers in phylogeographic studies, but they neither showed what markers were compared and why they chose the markers that they have used. Furthermore, phylogeographic studies to data have, however, faced extremely difficult to reveal the phylogeograpy of the species with extremely small populations (Gaos et al., 2016; Yang, Feng, & Gong, 2017). In this study, based on comparative plastomic approaches, two classes of molecular markers have been selected to inferring the genetic diversity or phylogeographic structure of *D*. *huoshanense*. According to our results, the phylogeograpy of *D*. *huoshanense* has been well resolved. Therefore, we put forth two implications for future phylogeographic studies: (a) Molecular marker screening is necessary before phylogeographic studies, (b) comparative plastomices approaches could revealed available molecular markers for phylogeographic study, especially for the species with extremely small populations.

## CONFLICT OF INTEREST

None declared.

## AUTHOR CONTRIBUTIONS

**Zhitao Niu:** Data curation (equal); formal analysis (equal); funding acquisition (equal); investigation (equal); software (equal); validation (equal); visualization (equal); writing‐original draft (lead); writing‐review & editing (lead). **Zhenyu Hou:** Data curation (equal); formal analysis (equal); software (equal). **Mengting Wang:** Data curation (equal); formal analysis (equal); validation (equal); visualization (equal). **Meirong Ye:** Validation (equal). **Benhou Zhang:** Data curation (equal); formal analysis (equal); investigation (equal); software (equal). **Qingyun Xue:** Writing‐review & editing (equal). **Wei Liu:** Data curation (equal); formal analysis (equal); investigation (equal). **Xiaoyu Ding:** Funding acquisition (equal); investigation (equal); supervision (equal); writing‐review & editing (equal).

D.X.Y. and N.Z.T.: Study design. N.Z.T., H.Z.Y., Y.M.R., and L.W.: Perform the experiments. N.Z.T., H.Z.Y., W.M.T, X.Q.Y., and Z.B.H.: Data analysis. N.Z.T.: Manuscript writing. All authors approved the final version of the manuscript.

## Supporting information

Figure S1

Figure S2

Figure S3

Figure S4

Table S1

Table S2

Table S3

Table S4

Table S5

## Data Availability

The DDBJ accession numbers for the two plastomes of *D*. *huoshanense* were LC493898 and LC493899, and DDBJ accession numbers for all chloroplast sequences LC494015–LC494038.

## References

[ece36277-bib-0001] Adams, P. B. (2011). Systematics of *Dendrobiinae* (Orchidaceae), with special reference to Australian taxa. Botanical Journal of the Linnean Society, 166, 105–126. 10.1111/j.1095-8339.2011.01141.x

[ece36277-bib-0002] Ahmed, I. , Matthews, P. J. , Biggs, P. J. , Naeem, M. , Mclenachan, P. A. , & Lockhart, P. J. (2013). Identification of chloroplast genome loci suitable for high‐resolution phylogeographic studies of *Colocasia esculenta* (L.) Schott (Araceae) and closely related taxa. Molecular Ecology Resources, 13, 929–937. 23718317 10.1111/1755-0998.12128

[ece36277-bib-0003] Avise, J. C. (2000). Phylogeography: The history and formation of species. Cambridge, MA, USA: Harvard University Press.

[ece36277-bib-0004] Bandelt, H. J. , Forster, P. , & Rohl, A. (1999). Median‐joining networks for inferring intraspecifc phylogenies. Molecular Biology and Evolution, 16, 37–48. 10331250 10.1093/oxfordjournals.molbev.a026036

[ece36277-bib-0005] Bao, X. S. , Shun, Q. S. , & Chen, L. Z. (2001). The medicinal plants of Dendrobium (Shi‐hu) in China. Shanghai, China: Shanghai Medicinal University Press and Fudan University Press.

[ece36277-bib-0006] Beheregaray, L. B. (2008). Twenty years of phylogeography: The state of the field and the challenges for the Southern Hemisphere. Molecular Ecology, 17, 3754–3774. 10.1111/j.1365-294X.2008.03857.x 18627447

[ece36277-bib-0007] Chase, M. W. , Cameron, K. M. , Barrett, R. L. , & Freudenstein, J. V. (2003). DNA data and Orchidaceae systematics: A new phylogenetic classification In Dixon K. W., Kell S. P., Barrett R. L., & Cribb P. J. (Eds.), Orchid conservation (pp. 69–89). Kota Kinabalu, Malaysia: Natural History Press.

[ece36277-bib-0008] Chen, Y. K. , Yang, X. B. , Yang, Q. , Li, D. H. , Long, W. X. , & Luo, W. Q. (2014). Factors affecting the distribution pattern of wild plants with extremely small populations in Hainan island. China. Plos ONE, 9, e97751 10.1371/journal.pone.0097751 24830683 PMC4022659

[ece36277-bib-0009] Cheng, Y. P. , Hwang, S. Y. , & Lin, T. P. (2005). Potential refugia in Taiwan revealed by the phylogeographical study of *Castanopsis carlesii* Hayata (Fagaceae). Molecular Ecology, 14, 2075–2085. 10.1111/j.1365-294X.2005.02567.x 15910328

[ece36277-bib-0010] Chung, M. Y. , & Chung, M. G. (2008). Conservation genetics of the endangered terrestrial orchid Pogonia minor in South Korea. Annales Botanici Fennici, 45, 455–464.

[ece36277-bib-0011] Doyle, J. J. , & Doyle, J. L. (1987). A rapid DNA isolation procedure for small quantities of fresh leaf tissue. Phytochemical Bulletin, 19, 11–15.

[ece36277-bib-0012] Earl, D. , Vonholdt, B. , Earl, D. A. , & VonHoldt, B. M. (2012). Structure Harvester: A website and program for visualizing STRUCTURE output and implementing the Evanno method. Conservation Genetics Resources, 4, 359–361. 10.1007/s12686-011-9548-7

[ece36277-bib-0013] Edgar, R. C. (2004). MUSCLE: Multiple sequence alignment with high accuracy and high throughput. Nucleic Acids Research, 32, 1792–1797. 10.1093/nar/gkh340 15034147 PMC390337

[ece36277-bib-0014] Excofer, L. , & Lischer, H. E. L. (2010). Arlequin suite ver 3.5: A new series of programs to perform population genetics analyses under Linux and Windows. Molecular Ecology Resources, 10, 564–567. 10.1111/j.1755-0998.2010.02847.x 21565059

[ece36277-bib-0015] Falush, D. , Stephens, M. , & Pritchard, J. K. (2003). Inference of population structure using multilocus genotype data: Linked loci and correlated allele frequencies. Genetics, 164, 1567–1587. 12930761 10.1093/genetics/164.4.1567 PMC1462648

[ece36277-bib-0016] Fay, M. F. , Pailler, T. , & Dixon, K. W. (2015). Orchid conservation: Making the links. Annals of Botany, 116, 377–379. 10.1093/aob/mcv142 26311710 PMC4549965

[ece36277-bib-0017] Gaos, A. R. , Lewison, R. L. , Liles, M. J. , Gadea, V. , Altamirano, E. , Henríquez, A. V. , … Dutton, P. H. (2016). Hawksbill turtle terra incognita: Conservation genetics of eastern Pacific rookeries. Ecology and Evolution, 6, 1251–1264. 26941950 10.1002/ece3.1897 PMC4761781

[ece36277-bib-0018] Ge, J. C. , Zha, X. Q. , Nie, C. Y. , Yu, N. J. , Li, Q. M. , Peng, D. Y. , … Luo, J. P. (2018). Polysaccharides from *Dendrobium huoshanense* stems alleviates lung inflammation in cigarette smoke‐induced mice. Carbohydrate Polymers, 189, 289–295. 10.1016/j.carbpol.2018.02.054 29580411

[ece36277-bib-0019] Guo, Y. Y. , Luo, Y. B. , Liu, Z. J. , & Wang, X. Q. (2015). Reticulate evolution and sea‐level fluctuations together drove species diversifcation of slipper orchids (*Paphiopedilum*) in South‐East Asia. Molecular Ecology, 24, 2838–2855. 25847454 10.1111/mec.13189

[ece36277-bib-0020] Hou, B. W. , Luo, J. , Zhang, Y. S. , Niu, Z. T. , Xue, Q. Y. , & Ding, X. Y. (2017). Iteration expansion and regional evolution: Phylogeography of *Dendrobium officinale* and four related taxa in southern China. Scientific Reports, 7, 43525 10.1038/srep43525 28262789 PMC5337965

[ece36277-bib-0021] Katoh, K. , & Standley, D. M. (2013). MAFFT multiple sequence alignment software version 7: Improvements in performance and usability. Molecular Biology and Evolution, 30, 772–780. 10.1093/molbev/mst010 23329690 PMC3603318

[ece36277-bib-0022] Li, Q. M. , Jiang, H. , Zha, X. Q. , Wu, D. L. , Pan, L. H. , Duan, J. , … Luo, J. P. (2018). Anti‐inflammatory bibenzyls from the stems of *Dendrobium huoshanense* via bioassay guided isolation. Natural Product Research, 3, 1–4. 10.1080/14786419.2018.1489394 30394105

[ece36277-bib-0023] Li, X. X. , Ding, X. Y. , Chu, B. H. , Zhou, Q. , Ding, G. , & Gu, S. (2008). Genetic diversity analysis and conservation of the endangered Chinese endemic herb *Dendrobium officinale* Kimura et Migo (Orchidaceae) based on AFLP. Genetica, 133, 159–166. 10.1007/s10709-007-9196-8 17805978

[ece36277-bib-0024] Li, Z. H. , Ma, X. , Wang, D. Y. , Li, Y. X. , Wang, C. W. , & Jin, X. H. (2019). Evolution of plastid genomes of *Holcoglossum* (Orchidaceae) with recent radiation. BMC Evolutionary Biology, 19, 63 10.1186/s12862-019-1384-5 30808310 PMC6390633

[ece36277-bib-0025] Liang, Z. Y. , Zhang, J. Y. , Huang, Y. C. , Zhou, C. J. , Wang, Y. W. , Zhou, C. H. , … Wei, G. (2019). Identification of flavonoids in *Dendrobium huoshanense* and comparison with those in allied species of *Dendrobium* by TLC, HPLC and HPLC coupled with electrospray ionization multi‐stage tandem MS analyses. Journal of Separation Science, 42, 1088–1104. 30663861 10.1002/jssc.201801021

[ece36277-bib-0026] Librado, P. , & Rozas, J. (2009). DnaSP v5: A software for comprehensive analysis of DNA polymorphism data. Bioinformatics, 25, 1451–1452. 10.1093/bioinformatics/btp187 19346325

[ece36277-bib-0027] Morris, A. B. , & Shaw, J. (2018). Markers in time and space: A review of the last decade of plant phylogeographic approaches. Molecular Ecology, 27, 2317–2333. 10.1111/mec.14695 29675939

[ece36277-bib-0028] Niu, Z. T. , Pan, J. J. , Xue, Q. Y. , Zhu, S. Y. , Liu, W. , & Ding, X. Y. (2018). Plastome‐wide comparison reveals new SNV resources for the authentication of *Dendrobium huoshanense* and its corresponding medicinal slice (Huoshan Fengdou). Acta Pharmaceutica Sinica B, 8, 466–477. 10.1016/j.apsb.2017.12.004 29881686 PMC5989833

[ece36277-bib-0029] Niu, Z. T. , Xue, Q. Y. , Zhu, S. Y. , Sun, J. , Liu, W. , & Ding, X. Y. (2017a). The complete plastome sequences of four orchid species: Insights into the evolution of the Orchidaceae and the utility of plastomic mutational hotspots. Frontiers in Plant Science, 8, 715 10.3389/fpls.2017.00715 28515737 PMC5413554

[ece36277-bib-0030] Niu, Z. T. , Zhu, S. Y. , Pan, J. J. , Li, L. D. , Sun, J. , & Ding, X. Y. (2017b). Comparative analysis of *Dendrobium* plastomes and utility of plastomic mutational hotspots. Scientific Reports, 7, 2073 10.1038/s41598-017-02252-8 28522861 PMC5437043

[ece36277-bib-0031] Phillips, R. D. , Dixon, K. W. , & Peakall, R. (2012). Low population genetic differentiation in the Orchidaceae: Implications for the diversification of the family. Molecular Ecology, 21, 5208–5220. 10.1111/mec.12036 23017205

[ece36277-bib-0032] Pinheiro, F. , Cafasso, D. , Cozzolino, S. , & Scopece, G. (2015). Transitions between self‐compatibility and self‐incompatibility and the evolution of reproductive isolation in the large and diverse tropical genus *Dendrobium* (Orchidaceae). Annals of Botany, 116, 457–467. 25953040 10.1093/aob/mcv057 PMC4549954

[ece36277-bib-0033] Pons, O. , & Petit, R. J. (1996). Measuring and testing genetic differentiation with ordered versus unordered alleles. Genetics, 144, 1237–1245. 8913764 10.1093/genetics/144.3.1237 PMC1207615

[ece36277-bib-0034] Posada, D. (2008). jModelTest: Phylogenetic model averaging. Molecular Biology and Evolution, 25, 1253–1256. 10.1093/molbev/msn083 18397919

[ece36277-bib-0035] Prince, L. M. (2015). Plastid primers for angiosperm phylogenetics and phylogeography. Applications in Plant Sciences, 3, 1400085 10.3732/apps.1400085 PMC4467757 26082876

[ece36277-bib-0036] Qiu, Y. X. , Fu, C. X. , & Comes, H. P. (2011). Plant molecular phylogeography in China and adjacent regions: Tracing the genetic imprints of Quaternary climate and environmental change in the world’s most diverse temperate flora. Molecular Phylogenetics and Evolution, 59, 225–244. 10.1016/j.ympev.2011.01.012 21292014

[ece36277-bib-0037] Ronquist, F. , Teslenko, M. , van der Mark, P. , Ayres, D. L. , Darling, A. , Höhna, S. , … Huelsenbeck, J. P. (2012). MrBayes 3.2: Efficient Bayesian phylogenetic inference and model choice across a large model space. Systematic Biology, 61, 539–542. 10.1093/sysbio/sys029 22357727 PMC3329765

[ece36277-bib-0038] Schattner, P. , Brooks, A. N. , & Lowe, T. M. (2005). The tRNAscan‐SE, snoscan and snoGPS web servers for the detection of tRNAs and snoRNAs. Nucleic Acids Research, 33, W686–689. 10.1093/nar/gki366 15980563 PMC1160127

[ece36277-bib-0039] Shaw, J. , Shafer, H. L. , Leonard, O. R. , Kovach, M. J. , Schorr, M. , & Morris, A. B. (2014). Chloroplast DNA sequence utility for the lowest phylogenetic and phylogeographic inferences in angiosperms: The tortoise and the hare IV. American Journal of Botany, 101, 1987–2004. 10.3732/ajb.1400398 25366863

[ece36277-bib-0040] Silvera, K. , Santiago, L. S. , Cushman, J. C. , & Winter, K. (2009). Crassulacean acid metabolism and epiphytism linked to adaptive radiations in the Orchidaceae. Plant Physiology, 149, 1838–1847. 10.1104/pp.108.132555 19182098 PMC2663729

[ece36277-bib-0041] State Forestry Administration of China . (2012). The saving and conservation program on extremely small populations in China. Beijing: State Forestry Administration of China.

[ece36277-bib-0042] Swarts, N. D. , & Dixon, K. W. (2009). Perspectives on orchid conservation in botanic gardens. Trends in Plant Science, 14, 590–598. 10.1016/j.tplants.2009.07.008 19733499

[ece36277-bib-0043] Tamura, K. , Peterson, D. , Peterson, N. , Stecher, G. , Nei, M. , & Kumar, S. (2011). MEGA5: Molecular evolutionary genetics analysis using maximum likelihood, evolutionary distance, and maximum parsimony methods. Molecular Biology and Evolution, 28, 2731–2739. 10.1093/molbev/msr121 21546353 PMC3203626

[ece36277-bib-0044] Tsi, Z. H. (1999). Flora Reipublicae Popularis Sinicae. Beijing: Science Press.

[ece36277-bib-0045] Twyford, A. D. , Kidner, C. A. , & Ennos, R. A. (2015). Maintenance of species boundaries in a Neotropical radiation of Begonia. Molecular Ecology, 24, 4982–4993. 26301313 10.1111/mec.13355 PMC4600226

[ece36277-bib-0046] Volis, S. (2016). How to conserve threatened Chinese plant species with extremely small populations? Plant Diversity, 38, 45–52. 10.1016/j.pld.2016.05.003 30159448 PMC6112090

[ece36277-bib-0047] Wang, L. Y. , Abbott, R. J. , Zheng, W. , Chen, P. , Wang, Y. J. , & Liu, J. Q. (2009). History and evolution of alpine plants endemic to the Qinghai‐Tibetan Plateau: *Aconitum gymnandrum* (Ranunculaceae). Molecular Ecology, 18, 709–721. 19175501 10.1111/j.1365-294X.2008.04055.x

[ece36277-bib-0048] Wang, X. , Lu, P. , & Luo, Z. (2013). GMATo: A novel tool for the identification and analysis of microsatellites in large genomes. Bioinformation, 9, 541–544. 10.6026/97320630009541 23861572 PMC3705631

[ece36277-bib-0049] Wood, H. P. (2006). The Dendrobiums. Portland, OR: Timber Press.

[ece36277-bib-0050] Wyman, S. K. , Jansen, R. K. , & Boore, J. L. (2004). Automatic annotation of organellar genomes with DOGMA. Bioinformatics, 20, 3252–3255. 10.1093/bioinformatics/bth352 15180927

[ece36277-bib-0051] Xie, S. Z. , Shang, Z. Z. , Li, Q. M. , Zha, X. Q. , Pan, L. H. , & Luo, J. P. (2019). *Dendrobium huoshanense* polysaccharide regulates intestinal lamina propria immune response by stimulation of intestinal epithelial cells via toll‐like receptor 4. Carbohydrate Polymers, 222, 115028 10.1016/j.carbpol.2019.115028 31320099

[ece36277-bib-0052] Xu, T. T. , Abbott, R. J. , Milne, R. I. , Mao, K. S. , Du, F. K. , Wu, G. L. et al (2010). Phylogeography and allopatric divergence of cypress species (*Cupressus* L.) in the Qinghai‐Tibetan Plateau and adjacent regions. BMC Evolutionary Biology, 10, 194. 20569425 10.1186/1471-2148-10-194 PMC3020627

[ece36277-bib-0053] Yang, J. B. , Tang, M. , Li, H. T. , Zhang, Z. R. , & Li, D. Z. (2013). Complete chloroplast genome of the genus *Cymbidium*: Lights into the species identification, phylogenetic implications and population genetic analyses. BMC Evolutionary Biology, 13, 84 10.1186/1471-2148-13-84 23597078 PMC3644226

[ece36277-bib-0054] Yang, R. , Feng, X. , & Gong, X. (2017). Genetic structure and demographic history of *Cycas chenii* (Cycadaceae), an endangered species with extremely small populations. Plant Diversity, 39, 44–51. 10.1016/j.pld.2016.11.003 30159490 PMC6112254

[ece36277-bib-0055] Yang, W. , Xiang, Z. , Zhang, S. , Kang, H. , & Shi, F. (2015). Plant species with extremely small populations (PSESP) and their significance in China's national plant conservation strategy. Biodiversity Science, 23, 419–425.

[ece36277-bib-0056] Ye, M. R. , Liu, W. , Xue, Q. Y. , Hou, B. W. , Luo, J. , & Ding, X. Y. (2016). Phylogeography of the endangered orchid *Dendrobium moniliforme* in East Asia inferred from chloroplast DNA sequences. Mitochondrial DNA Part A, 12, 17–29. 10.1080/24701394.2016.1202942 27931140

