# Supplementary material for: A comparative plastomics approach reveals available molecular markers for the phylogeographic study of Dendrobium huoshanense, an endangered orchid with extremely small populations
Source: Ecol Evol. 2020 Apr 30;10(12):5332–42. doi: 10.1002/ece3.6277 (PMC7319108; doi:10.1002/ece3.6277)
Supplement: Supplementary file 1 — Figure S1 [file ECE3-10-5332-s001.pdf]

Figure S1

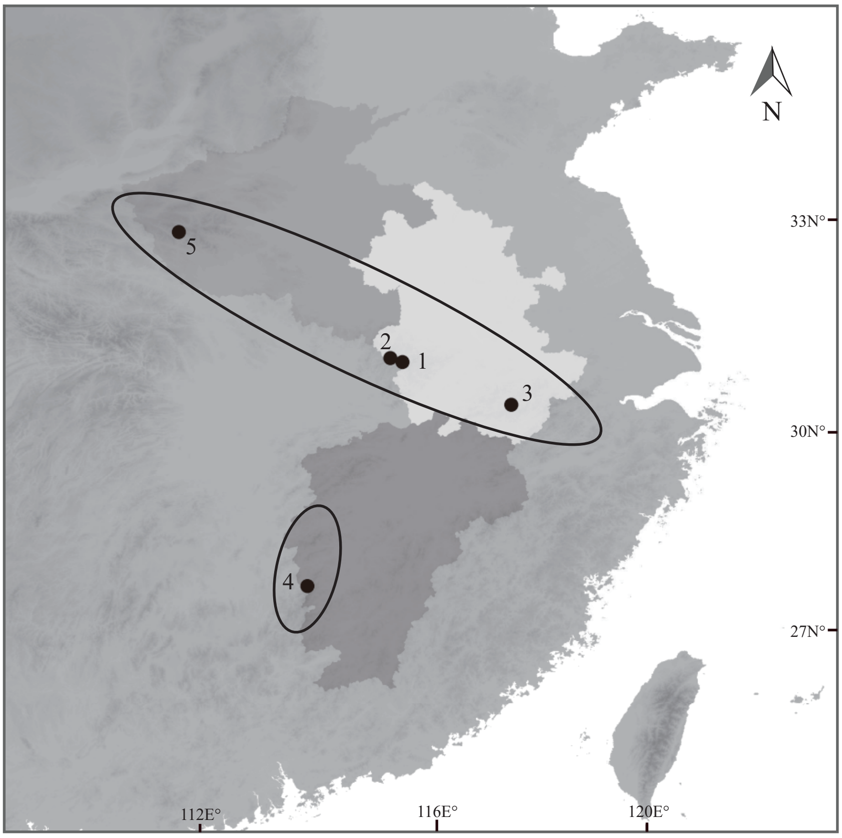

Geographical distribution of *Dendrobium huoshanense*

Sampling locations of *Dendrobium huoshanense*
