# Supplementary figures and images for: A comparative plastomics approach reveals available molecular markers for the phylogeographic study of Dendrobium huoshanense, an endangered orchid with extremely small populations
Source: Ecol Evol. 2020 Apr 30;10(12):5332–42. doi: 10.1002/ece3.6277 (PMC7319108; doi:10.1002/ece3.6277)

Figure S2

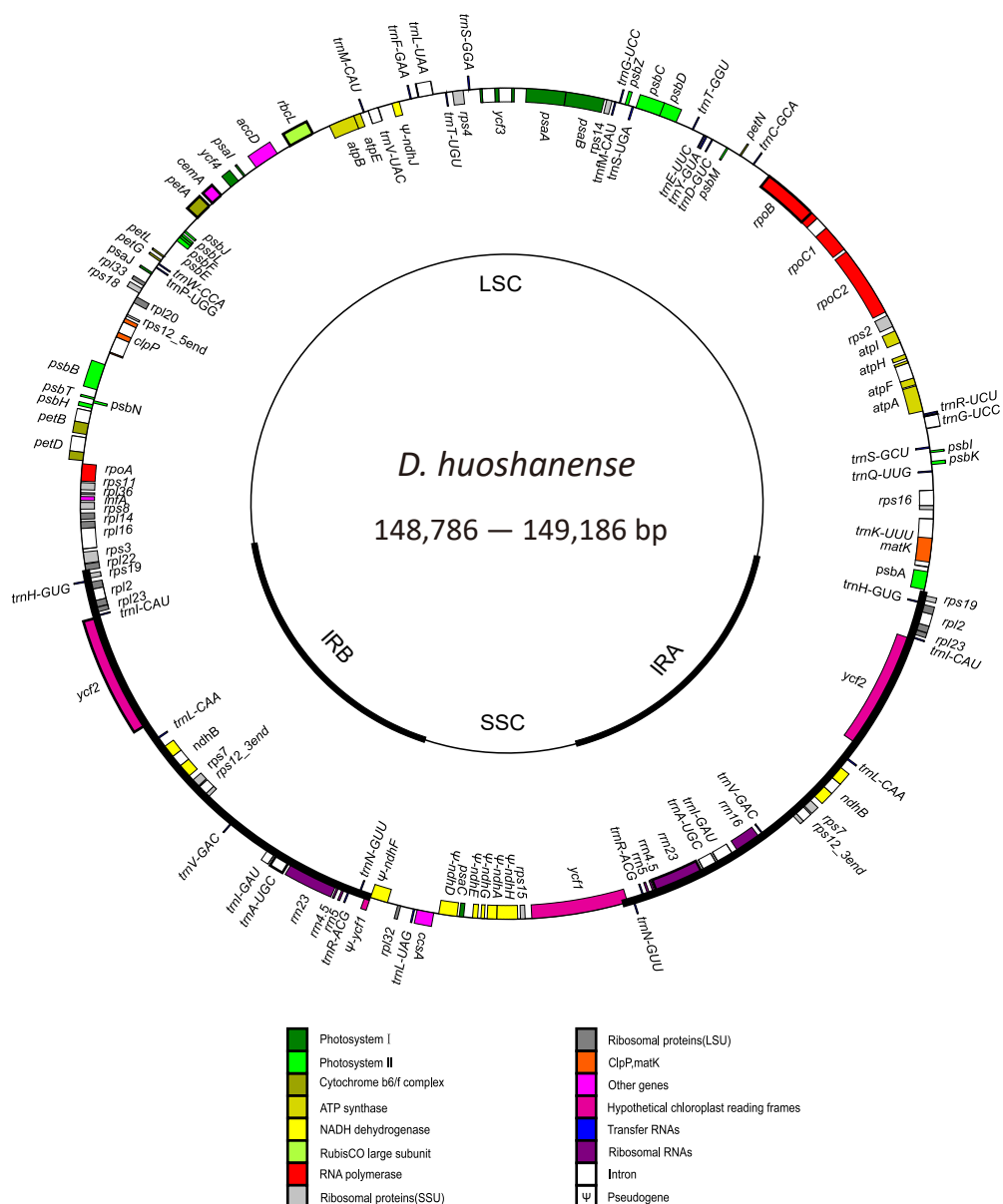

Supplement: Supplementary file 2 — Figure S2 [file ECE3-10-5332-s002.pdf]

Figure S3

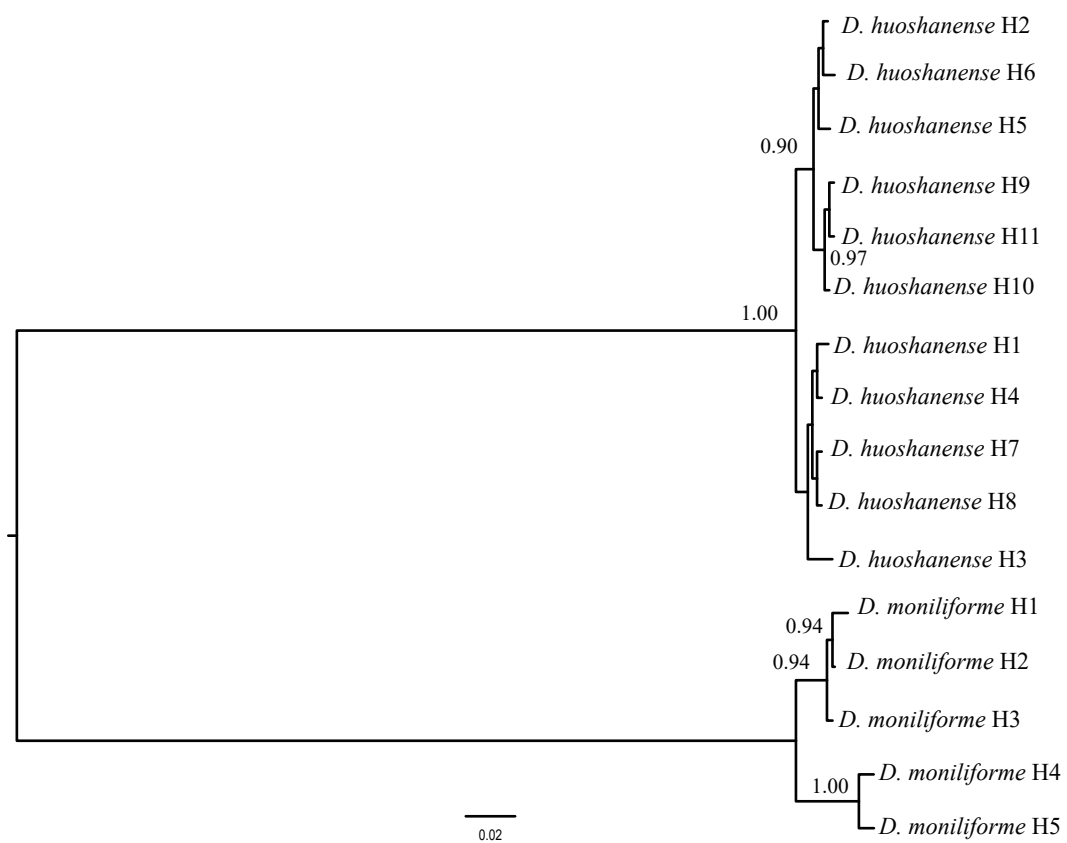

Supplement: Supplementary file 3 — Figure S3 [file ECE3-10-5332-s003.pdf]

Figure S4

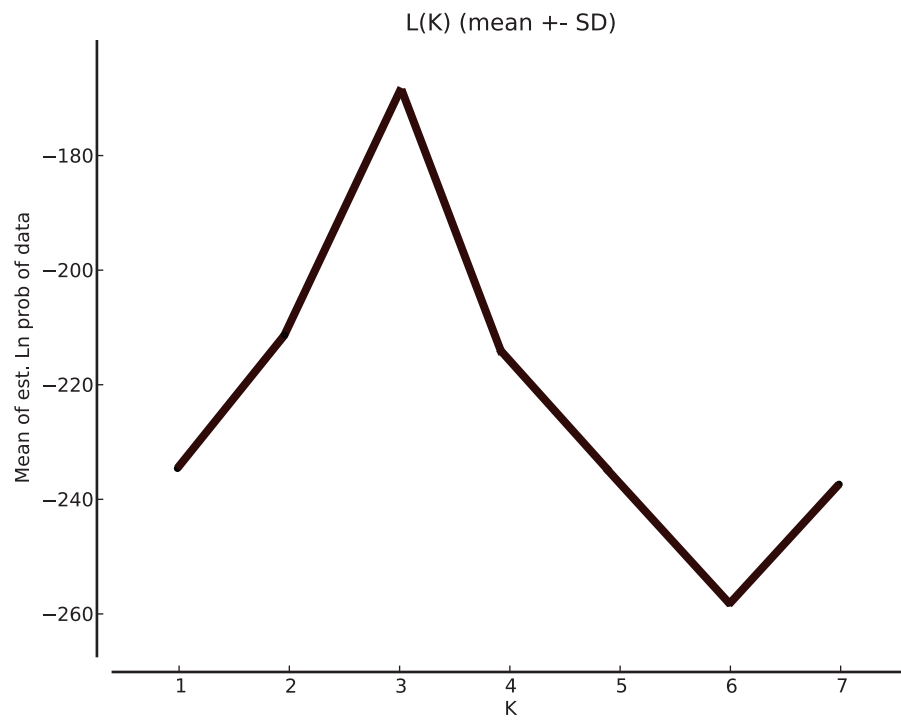

Supplement: Supplementary file 4 — Figure S4 [file ECE3-10-5332-s004.pdf]
