# Supplementary material for: A comparative plastomics approach reveals available molecular markers for the phylogeographic study of Dendrobium huoshanense, an endangered orchid with extremely small populations
Source: Ecol Evol. 2020 Apr 30;10(12):5332–42. doi: 10.1002/ece3.6277 (PMC7319108; doi:10.1002/ece3.6277)
Supplement: Supplementary file 5 — Table S1 [file ECE3-10-5332-s005.docx]

| Table S1 Primers used to amplify 27 hotspot and SC-IR regions | | | |
| --- | --- | --- | --- |
| Loci | Primers (5'-3') | | Tm (℃) |
| Hotspot 9 | CTGAAGGAGTAGCGGATATAGGAAG | CGGGTCTAGTGAATAAATGGATAGAG | 54 |
| Hotspot 10 | CTGAAGGAGTAGCGGATATAGG | GGCTCCAATTCGAGTAAGAC | 52 |
| Hotspot 12 | CTACCGTTGAGTTAGCAACCCGGATC | GAGCCATCTATCGAATCGTTGC | 53 |
| Hotspot 16 | GTCAGAATCCACATCCAATCATGTCCTTC | GAATCCCAGCGACAACATGTCCTATATTTC | 59 |
| Hotspot 17 | CAGAATCCACATCCAATCATGTCC | GAATCCCAGCGACAACATG | 56 |
| Hotspot 19 | GTAAGGAAGGATTCTTGATTGGGGAG | CTAATCCGTTGTACGAATTATTCGTACC | 57 |
| Hotspot 20 |  |  |  |
| Hotspot 22 | CAGAATCCACATCCAATCATGTCC | GAATCCTTTCGTCCCAGATCGTTTGCAT | 55 |
| Hotspot 24 | CTGGCGGTTCAAGTGAAGGGAAG | GCTTCTCGACTCCATAATCCAAATT | 60 |
| Hotspot 69 | CGGAAGCAAGACAGAGAAATGGAG | CAAGGATTCGTCAAGTTCGATCAAC | 58 |
| Hotspot 70 |  |  |  |
| Hotspot 71 | GTTGATCGAACTTGACGAATCCTTG | CCAAGCGAGACTTACTATATCCATGTG | 57 |
| Hotspot 72 |  |  |  |
| Hotspot 73 | GTATCCAGACTCACTAGAGGCTCTG | CTGAGATAGTATGGTAGAAAGAGCTAT | 53 |
| Hotspot 74 |  |  |  |
| Hotspot 124 | GAGTGGAAGGATAGGAATAGAAAGG | GATTGCCGAGGAATAGAGACTTTAC | 53 |
| Hotspot 125 |  |  |  |
| Hotspot 126 | CTCTATTCCTCGGCAATCAAAGC | CTTACTCCGTAGATTCGAGAGAACAAG | 56 |
| Hotspot 127 |  |  |  |
| Hotspot 128 |  |  |  |
| Hotspot 180 | GGTCAAATGATTCCAGTACCACTTC | CAGTATGAACACGATACCAAGGC | 55 |
| Hotspot 181 |  |  |  |
| Hotspot 182 |  |  |  |
| Hotspot 252 | CTCAGACCAATTCATTCACGTTT | GACCTCCTTGCTTCTCTCATGGTAC | 53 |
| Hotspot 253 | CTCCTCCCTTCTTCTACTCCGTCC | GTTGACCTCCTTGCTTCTCTCATG | 58 |
| Hotspot 254 | CTTCCGTGATGAACTGTTGGCAC | GACCGGGAAGGGATATAACTCAGC | 58 |
| Hotspot 255 | CGTGATGAACTGTTGGCACCAGTC | CGAACTGATGACTTCCACCACGTC | 60 |
| LSC-IR_A_ | CCGATTGATCTTCCAATATGCTAGTT | CTTGGTCTCGGGCATCTACCATTAT | 57 |
| LSC-IR_B_ | CTCCCAATTTGTGACCGACCATA | CCGGATCTAAGTGTTGGCTAGGTAAG | 58 |
| IR_B_-SSC | CCTACGACCAGTCAGTTAACAGCC | CTCGAACCGAGATGCTCTAGCAC | 56 |
| IR_A_-SSC | CCGTATCCAGAATAATACCAATCCA | CCTACGACCAGTCAGTTAACAGCC | 56 |
|  | | | |
