# Supplementary material for: A comparative plastomics approach reveals available molecular markers for the phylogeographic study of Dendrobium huoshanense, an endangered orchid with extremely small populations
Source: Ecol Evol. 2020 Apr 30;10(12):5332–42. doi: 10.1002/ece3.6277 (PMC7319108; doi:10.1002/ece3.6277)
Supplement: Supplementary file 6 — Table S2 [file ECE3-10-5332-s006.docx]

| Tabel S2. The estimated GC content and the number of SNPs, InDels and cpSSRs for the 372 bins. | | | | |
| --- | --- | --- | --- | --- |
| Bins | GC content | Number of SNPs | Number of InDels | Number of cpSSRs |
| 1 | 40.04% | 0.33 | 0.83 | 0 |
| 2 | 43.56% | 0.50 | 0.00 | 0 |
| 3 | 40.75% | 0.00 | 0.00 | 0 |
| 4 | 33.00% | 0.00 | 0.00 | 1 |
| 5 | 31.94% | 0.50 | 0.00 | 1 |
| 6 | 33.06% | 0.50 | 0.00 | 0 |
| 7 | 32.00% | 0.00 | 0.00 | 1 |
| 8 | 31.00% | 0.00 | 0.00 | 0 |
| 9 | 25.30% | 6.50 | 1.83 | 3 |
| 10 | 30.57% | 4.50 | 2.33 | 1 |
| 11 | 30.88% | 1.00 | 0.00 | 2 |
| 12 | 26.62% | 5.00 | 3.50 | 3 |
| 13 | 32.19% | 3.50 | 0.00 | 0 |
| 14 | 33.00% | 0.00 | 0.00 | 1 |
| 15 | 33.69% | 1.00 | 0.00 | 0 |
| 16 | 25.05% | 4.00 | 2.00 | 1 |
| 17 | 22.50% | 1.00 | 1.33 | 1 |
| 18 | 29.63% | 1.00 | 0.00 | 0 |
| 19 | 31.11% | 1.00 | 1.00 | 1 |
| 20 | 29.47% | 0.00 | 2.00 | 2 |
| 21 | 37.00% | 0.00 | 0.00 | 1 |
| 22 | 21.48% | 3.00 | 2.50 | 0 |
| 23 | 24.64% | 1.17 | 0.00 | 0 |
| 24 | 29.38% | 1.67 | 2.33 | 1 |
| 25 | 33.75% | 0.00 | 0.00 | 0 |
| 26 | 34.66% | 0.50 | 1.50 | 1 |
| 27 | 36.42% | 0.00 | 0.50 | 0 |
| 28 | 38.75% | 0.00 | 0.00 | 0 |
| 29 | 42.83% | 0.00 | 0.67 | 0 |
| 30 | 37.81% | 1.00 | 0.00 | 0 |
| 31 | 33.75% | 0.00 | 0.00 | 1 |
| 32 | 28.44% | 0.50 | 0.00 | 1 |
| 33 | 30.95% | 0.17 | 1.00 | 0 |
| 34 | 34.50% | 0.00 | 0.00 | 1 |
| 35 | 38.75% | 0.00 | 0.00 | 0 |
| 36 | 29.93% | 0.50 | 1.50 | 1 |
| 37 | 35.10% | 0.00 | 1.17 | 0 |
| 38 | 38.13% | 1.00 | 0.00 | 0 |
| 39 | 28.28% | 1.50 | 1.33 | 1 |
| 40 | 38.25% | 0.00 | 0.00 | 0 |
| 41 | 36.07% | 0.00 | 0.50 | 1 |
| 42 | 35.25% | 1.00 | 0.00 | 0 |
| 43 | 41.75% | 0.00 | 0.00 | 0 |
| 44 | 35.81% | 0.50 | 0.00 | 0 |
| 45 | 32.63% | 1.00 | 0.00 | 0 |
| 46 | 35.69% | 0.50 | 0.00 | 1 |
| 47 | 35.56% | 0.50 | 0.00 | 2 |
| 48 | 33.50% | 0.00 | 0.50 | 0 |
| 49 | 36.25% | 0.00 | 0.00 | 0 |
| 50 | 41.25% | 0.00 | 0.00 | 1 |
| 51 | 41.00% | 0.00 | 0.00 | 0 |
| 52 | 38.11% | 1.00 | 0.50 | 0 |
| 53 | 37.68% | 1.00 | 0.50 | 1 |
| 54 | 40.25% | 0.00 | 0.00 | 0 |
| 55 | 41.00% | 2.00 | 0.00 | 0 |
| 56 | 38.00% | 0.00 | 0.00 | 0 |
| 57 | 36.82% | 0.00 | 0.50 | 1 |
| 58 | 38.70% | 1.00 | 0.50 | 1 |
| 59 | 38.56% | 0.50 | 0.00 | 0 |
| 60 | 40.19% | 0.50 | 0.00 | 0 |
| 61 | 41.19% | 0.50 | 0.00 | 0 |
| 62 | 39.75% | 0.00 | 0.00 | 0 |
| 63 | 37.50% | 0.00 | 0.00 | 0 |
| 64 | 40.75% | 0.00 | 0.00 | 0 |
| 65 | 40.75% | 0.50 | 0.00 | 0 |
| 66 | 34.44% | 0.50 | 0.00 | 1 |
| 67 | 35.00% | 0.00 | 0.00 | 0 |
| 68 | 35.50% | 0.00 | 0.00 | 0 |
| 69 | 30.15% | 4.00 | 1.17 | 0 |
| 70 | 28.95% | 0.00 | 0.50 | 0 |
| 71 | 40.39% | 1.00 | 0.50 | 1 |
| 72 | 30.11% | 1.00 | 2.00 | 2 |
| 73 | 30.20% | 0.50 | 1.00 | 1 |
| 74 | 27.73% | 0.50 | 1.67 | 0 |
| 75 | 29.19% | 0.50 | 0.00 | 0 |
| 76 | 29.25% | 3.00 | 0.00 | 0 |
| 77 | 38.56% | 0.50 | 0.00 | 0 |
| 78 | 36.00% | 0.00 | 0.00 | 1 |
| 79 | 37.42% | 0.50 | 0.50 | 1 |
| 80 | 29.92% | 1.50 | 0.83 | 1 |
| 81 | 35.89% | 1.50 | 0.50 | 1 |
| 82 | 31.69% | 0.50 | 0.00 | 0 |
| 83 | 35.35% | 1.00 | 1.00 | 0 |
| 84 | 42.56% | 0.50 | 0.00 | 0 |
| 85 | 43.25% | 0.00 | 0.00 | 0 |
| 86 | 45.75% | 0.00 | 0.00 | 0 |
| 87 | 41.00% | 0.00 | 0.00 | 0 |
| 88 | 44.00% | 0.00 | 0.00 | 0 |
| 89 | 43.78% | 0.17 | 0.00 | 0 |
| 90 | 38.64% | 0.17 | 1.17 | 0 |
| 91 | 36.75% | 0.00 | 0.00 | 1 |
| 92 | 32.25% | 0.50 | 0.50 | 1 |
| 93 | 41.50% | 0.00 | 0.00 | 0 |
| 94 | 38.81% | 0.50 | 0.00 | 0 |
| 95 | 37.75% | 0.00 | 0.00 | 0 |
| 96 | 39.50% | 0.00 | 0.00 | 0 |
| 97 | 40.75% | 0.00 | 0.00 | 0 |
| 98 | 41.81% | 0.50 | 0.00 | 0 |
| 99 | 42.85% | 0.50 | 0.67 | 0 |
| 100 | 42.44% | 0.50 | 0.00 | 0 |
| 101 | 42.00% | 0.00 | 0.00 | 0 |
| 102 | 44.00% | 0.00 | 0.00 | 1 |
| 103 | 42.25% | 0.00 | 0.00 | 0 |
| 104 | 43.75% | 0.00 | 0.00 | 0 |
| 105 | 43.50% | 0.00 | 0.00 | 0 |
| 106 | 40.00% | 0.00 | 0.00 | 0 |
| 107 | 26.56% | 0.50 | 0.00 | 0 |
| 108 | 37.94% | 0.50 | 0.00 | 1 |
| 109 | 31.50% | 0.00 | 0.00 | 0 |
| 110 | 35.00% | 0.00 | 0.00 | 0 |
| 111 | 38.69% | 0.50 | 0.00 | 0 |
| 112 | 34.75% | 0.00 | 0.00 | 2 |
| 113 | 30.94% | 0.50 | 0.00 | 0 |
| 114 | 36.50% | 1.00 | 0.00 | 0 |
| 115 | 33.88% | 1.00 | 0.00 | 0 |
| 116 | 40.25% | 0.00 | 0.00 | 0 |
| 117 | 32.69% | 0.50 | 0.00 | 0 |
| 118 | 21.35% | 5.67 | 3.33 | 2 |
| 119 | 35.31% | 0.50 | 0.00 | 1 |
| 120 | 29.93% | 1.00 | 0.50 | 1 |
| 121 | 28.38% | 1.00 | 0.00 | 0 |
| 122 | 30.79% | 0.50 | 1.00 | 2 |
| 123 | 31.75% | 0.00 | 0.00 | 1 |
| 124 | 37.56% | 3.17 | 1.33 | 0 |
| 125 | 36.20% | 1.00 | 0.50 | 0 |
| 126 | 24.97% | 1.00 | 1.83 | 2 |
| 127 | 36.23% | 0.50 | 0.50 | 0 |
| 128 | 39.85% | 1.00 | 0.50 | 0 |
| 129 | 37.13% | 1.00 | 0.00 | 0 |
| 130 | 35.25% | 0.00 | 0.00 | 0 |
| 131 | 42.75% | 0.00 | 0.00 | 0 |
| 132 | 42.75% | 0.00 | 0.00 | 0 |
| 133 | 44.75% | 0.00 | 0.00 | 0 |
| 134 | 42.25% | 0.00 | 0.00 | 0 |
| 135 | 32.25% | 1.50 | 0.50 | 1 |
| 136 | 18.46% | 2.17 | 0.83 | 2 |
| 137 | 29.00% | 0.00 | 0.00 | 0 |
| 138 | 44.66% | 0.00 | 0.67 | 0 |
| 139 | 41.50% | 0.00 | 0.00 | 0 |
| 140 | 45.50% | 0.00 | 0.00 | 0 |
| 141 | 34.84% | 0.50 | 0.50 | 0 |
| 142 | 32.66% | 0.50 | 1.00 | 0 |
| 143 | 30.25% | 0.00 | 0.00 | 0 |
| 144 | 28.56% | 0.50 | 0.00 | 0 |
| 145 | 34.06% | 0.50 | 0.00 | 0 |
| 146 | 39.25% | 0.00 | 0.00 | 0 |
| 147 | 30.13% | 0.67 | 0.83 | 0 |
| 148 | 28.28% | 5.83 | 0.83 | 0 |
| 149 | 31.36% | 3.00 | 1.50 | 1 |
| 150 | 38.13% | 1.00 | 0.00 | 0 |
| 151 | 38.94% | 0.50 | 0.00 | 0 |
| 152 | 32.76% | 0.50 | 0.50 | 0 |
| 153 | 33.44% | 0.50 | 0.00 | 1 |
| 154 | 35.00% | 0.00 | 0.00 | 0 |
| 155 | 29.25% | 0.00 | 0.00 | 0 |
| 156 | 39.88% | 1.00 | 0.00 | 1 |
| 157 | 41.03% | 0.00 | 0.50 | 0 |
| 158 | 28.91% | 2.50 | 0.50 | 1 |
| 159 | 29.00% | 0.00 | 0.00 | 1 |
| 160 | 34.81% | 0.50 | 0.00 | 0 |
| 161 | 36.25% | 0.00 | 0.00 | 0 |
| 162 | 39.50% | 0.00 | 0.00 | 1 |
| 163 | 29.99% | 0.00 | 0.67 | 1 |
| 164 | 25.94% | 0.50 | 0.00 | 1 |
| 165 | 29.38% | 1.00 | 0.00 | 1 |
| 166 | 35.25% | 0.00 | 0.00 | 0 |
| 167 | 36.50% | 0.00 | 0.00 | 0 |
| 168 | 29.44% | 0.50 | 0.00 | 2 |
| 169 | 26.46% | 1.50 | 1.00 | 0 |
| 170 | 32.75% | 0.00 | 0.00 | 0 |
| 171 | 32.88% | 0.50 | 0.50 | 1 |
| 172 | 29.20% | 0.00 | 1.00 | 1 |
| 173 | 37.66% | 0.00 | 0.50 | 0 |
| 174 | 37.31% | 0.50 | 0.00 | 0 |
| 175 | 36.70% | 0.00 | 0.50 | 0 |
| 176 | 36.50% | 0.00 | 0.00 | 0 |
| 177 | 34.00% | 0.00 | 0.00 | 0 |
| 178 | 36.26% | 0.50 | 0.50 | 2 |
| 179 | 36.31% | 0.50 | 0.00 | 0 |
| 180 | 23.32% | 1.00 | 1.00 | 1 |
| 181 | 35.57% | 0.50 | 0.50 | 0 |
| 182 | 25.07% | 9.50 | 2.67 | 3 |
| 183 | 42.00% | 0.00 | 0.00 | 0 |
| 184 | 44.90% | 0.00 | 0.83 | 1 |
| 185 | 44.63% | 1.00 | 0.00 | 0 |
| 186 | 42.75% | 0.00 | 0.00 | 0 |
| 187 | 18.56% | 1.00 | 1.33 | 0 |
| 188 | 35.63% | 0.50 | 0.50 | 0 |
| 189 | 32.44% | 0.50 | 0.00 | 0 |
| 190 | 31.32% | 5.00 | 0.50 | 0 |
| 191 | 38.18% | 0.00 | 0.50 | 1 |
| 192 | 41.50% | 0.00 | 0.00 | 0 |
| 193 | 33.94% | 0.50 | 0.00 | 0 |
| 194 | 35.57% | 0.50 | 0.50 | 1 |
| 195 | 32.75% | 0.67 | 1.00 | 0 |
| 196 | 37.50% | 0.00 | 0.00 | 0 |
| 197 | 32.63% | 0.00 | 0.50 | 1 |
| 198 | 32.75% | 0.00 | 0.00 | 0 |
| 199 | 38.00% | 1.50 | 0.00 | 0 |
| 200 | 41.00% | 0.00 | 0.00 | 0 |
| 201 | 35.27% | 0.50 | 0.50 | 0 |
| 202 | 35.07% | 0.00 | 0.50 | 1 |
| 203 | 35.00% | 0.00 | 0.00 | 0 |
| 204 | 27.46% | 1.00 | 0.50 | 1 |
| 205 | 37.45% | 0.00 | 0.50 | 0 |
| 206 | 42.19% | 0.50 | 0.00 | 0 |
| 207 | 30.06% | 1.00 | 0.00 | 1 |
| 208 | 31.69% | 0.50 | 0.00 | 0 |
| 209 | 27.63% | 0.00 | 1.00 | 1 |
| 210 | 30.97% | 0.00 | 0.50 | 0 |
| 211 | 31.57% | 3.67 | 0.50 | 1 |
| 212 | 31.06% | 1.00 | 0.00 | 2 |
| 213 | 35.25% | 0.00 | 0.00 | 0 |
| 214 | 39.25% | 0.00 | 0.00 | 1 |
| 215 | 45.00% | 0.00 | 0.00 | 0 |
| 216 | 41.43% | 0.00 | 1.00 | 0 |
| 217 | 41.25% | 0.00 | 0.00 | 0 |
| 218 | 40.90% | 0.00 | 0.50 | 0 |
| 219 | 37.25% | 0.00 | 0.00 | 0 |
| 220 | 38.00% | 0.00 | 0.00 | 0 |
| 221 | 40.75% | 0.00 | 0.00 | 0 |
| 222 | 41.00% | 0.00 | 0.00 | 0 |
| 223 | 38.07% | 0.00 | 0.67 | 0 |
| 224 | 39.50% | 0.00 | 0.00 | 0 |
| 225 | 36.81% | 0.50 | 0.00 | 0 |
| 226 | 36.71% | 0.00 | 0.67 | 0 |
| 227 | 35.25% | 0.00 | 0.00 | 0 |
| 228 | 36.75% | 0.00 | 0.00 | 0 |
| 229 | 38.00% | 0.00 | 0.00 | 0 |
| 230 | 39.75% | 0.00 | 0.00 | 0 |
| 231 | 35.00% | 0.00 | 0.00 | 0 |
| 232 | 38.75% | 0.00 | 0.00 | 0 |
| 233 | 37.75% | 0.00 | 0.00 | 0 |
| 234 | 38.75% | 0.00 | 0.00 | 0 |
| 235 | 39.86% | 0.50 | 0.50 | 0 |
| 236 | 43.00% | 0.00 | 0.00 | 0 |
| 237 | 39.00% | 0.00 | 0.00 | 0 |
| 238 | 42.67% | 0.50 | 0.50 | 0 |
| 239 | 46.00% | 0.00 | 0.00 | 0 |
| 240 | 38.74% | 0.00 | 0.50 | 0 |
| 241 | 38.50% | 0.00 | 0.00 | 0 |
| 242 | 38.50% | 0.00 | 0.00 | 0 |
| 243 | 39.75% | 0.00 | 0.00 | 0 |
| 244 | 39.50% | 0.00 | 0.00 | 0 |
| 245 | 41.25% | 0.00 | 0.00 | 0 |
| 246 | 34.50% | 0.00 | 0.00 | 0 |
| 247 | 35.00% | 0.00 | 0.00 | 0 |
| 248 | 41.50% | 0.00 | 0.00 | 0 |
| 249 | 38.00% | 0.00 | 0.00 | 0 |
| 250 | 42.50% | 0.00 | 0.00 | 0 |
| 251 | 39.56% | 0.50 | 0.00 | 0 |
| 252 | 36.48% | 23.00 | 2.67 | 2 |
| 253 | 34.72% | 0.00 | 1.50 | 0 |
| 254 | 43.45% | 0.00 | 0.50 | 0 |
| 255 | 40.63% | 0.50 | 1.00 | 2 |
| 256 | 47.19% | 0.50 | 0.00 | 0 |
| 257 | 56.41% | 0.00 | 0.00 | 0 |
| 258 | 54.50% | 0.00 | 0.00 | 0 |
| 259 | 55.98% | 0.00 | 0.50 | 0 |
| 260 | 53.45% | 2.00 | 0.67 | 1 |
| 261 | 54.25% | 0.00 | 0.00 | 0 |
| 262 | 50.25% | 0.00 | 0.00 | 1 |
| 263 | 48.00% | 0.00 | 0.00 | 0 |
| 264 | 49.75% | 0.00 | 0.00 | 0 |
| 265 | 52.00% | 0.00 | 0.00 | 0 |
| 266 | 50.59% | 0.00 | 0.50 | 0 |
| 267 | 52.93% | 0.50 | 0.50 | 0 |
| 268 | 54.00% | 0.00 | 0.00 | 0 |
| 269 | 56.00% | 0.00 | 0.00 | 0 |
| 270 | 54.25% | 0.00 | 0.00 | 0 |
| 271 | 58.42% | 0.00 | 0.67 | 0 |
| 272 | 56.25% | 0.00 | 0.00 | 0 |
| 273 | 54.44% | 3.17 | 0.83 | 0 |
| 274 | 44.00% | 0.00 | 0.00 | 0 |
| 275 | 50.32% | 0.00 | 1.00 | 0 |
| 276 | 42.75% | 0.00 | 0.00 | 0 |
| 277 | 37.75% | 0.00 | 0.00 | 0 |
| 278 | 32.14% | 1.00 | 1.00 | 0 |
| 279 | 12.84% | 21.17 | 4.50 | 4 |
| 280 | 26.25% | 0.00 | 0.00 | 2 |
| 281 | 23.64% | 1.17 | 1.17 | 1 |
| 282 | 31.94% | 1.00 | 1.33 | 1 |
| 283 | 28.56% | 2.00 | 0.00 | 0 |
| 284 | 32.50% | 0.00 | 0.00 | 0 |
| 285 | 33.00% | 0.00 | 0.00 | 0 |
| 286 | 33.50% | 0.00 | 1.17 | 2 |
| 287 | 37.75% | 0.00 | 0.00 | 0 |
| 288 | 37.00% | 0.00 | 0.00 | 0 |
| 289 | 33.00% | 0.00 | 0.00 | 0 |
| 290 | 24.24% | 0.00 | 0.50 | 0 |
| 291 | 29.25% | 1.00 | 0.00 | 0 |
| 292 | 30.30% | 0.50 | 0.50 | 2 |
| 293 | 26.25% | 1.00 | 0.00 | 0 |
| 294 | 29.06% | 0.50 | 0.00 | 0 |
| 295 | 30.44% | 0.50 | 0.00 | 1 |
| 296 | 27.31% | 0.50 | 0.00 | 1 |
| 297 | 27.81% | 1.50 | 0.67 | 2 |
| 298 | 27.00% | 0.50 | 0.00 | 1 |
| 299 | 25.48% | 1.00 | 0.50 | 0 |
| 300 | 26.57% | 4.00 | 1.00 | 1 |
| 301 | 28.75% | 1.00 | 0.00 | 3 |
| 302 | 29.81% | 1.50 | 0.00 | 1 |
| 303 | 33.72% | 12.50 | 1.67 | 1 |
| 304 | 28.18% | 2.83 | 1.00 | 3 |
| 305 | 32.69% | 0.50 | 0.00 | 1 |
| 306 | 36.69% | 0.50 | 0.00 | 0 |
| 307 | 38.19% | 0.50 | 0.00 | 0 |
| 308 | 36.00% | 0.00 | 0.00 | 0 |
| 309 | 42.75% | 0.00 | 0.00 | 0 |
| 310 | 50.57% | 0.00 | 1.00 | 0 |
| 311 | 45.50% | 0.00 | 0.00 | 0 |
| 312 | 52.28% | 2.00 | 0.83 | 0 |
| 313 | 57.50% | 0.00 | 0.00 | 0 |
| 314 | 57.22% | 0.00 | 0.67 | 0 |
| 315 | 54.75% | 0.00 | 0.00 | 0 |
| 316 | 56.25% | 0.00 | 0.00 | 0 |
| 317 | 53.25% | 0.00 | 0.00 | 0 |
| 318 | 54.92% | 0.50 | 0.50 | 0 |
| 319 | 48.60% | 0.00 | 0.50 | 0 |
| 320 | 52.25% | 0.00 | 0.00 | 0 |
| 321 | 50.00% | 0.00 | 0.00 | 0 |
| 322 | 49.00% | 0.00 | 0.00 | 0 |
| 323 | 47.50% | 0.00 | 0.00 | 0 |
| 324 | 56.75% | 0.00 | 0.00 | 1 |
| 325 | 52.48% | 2.00 | 0.67 | 0 |
| 326 | 57.50% | 0.00 | 0.00 | 0 |
| 327 | 53.57% | 0.00 | 0.50 | 0 |
| 328 | 56.16% | 0.00 | 0.50 | 0 |
| 329 | 48.69% | 0.50 | 0.00 | 0 |
| 330 | 40.39% | 0.50 | 1.00 | 2 |
| 331 | 43.45% | 0.00 | 0.50 | 0 |
| 332 | 34.72% | 0.00 | 1.50 | 0 |
| 333 | 36.27% | 23.67 | 2.83 | 1 |
| 334 | 40.81% | 0.50 | 0.00 | 0 |
| 335 | 41.75% | 0.00 | 0.00 | 0 |
| 336 | 38.00% | 0.00 | 0.00 | 0 |
| 337 | 41.25% | 0.00 | 0.00 | 0 |
| 338 | 36.75% | 0.00 | 0.00 | 0 |
| 339 | 34.00% | 0.00 | 0.00 | 0 |
| 340 | 41.25% | 0.00 | 0.00 | 0 |
| 341 | 38.25% | 0.00 | 0.00 | 0 |
| 342 | 40.50% | 0.00 | 0.00 | 0 |
| 343 | 39.50% | 0.00 | 0.00 | 0 |
| 344 | 37.50% | 0.00 | 0.00 | 0 |
| 345 | 37.49% | 0.00 | 0.50 | 0 |
| 346 | 46.50% | 0.00 | 0.00 | 0 |
| 347 | 43.92% | 0.50 | 0.50 | 0 |
| 348 | 39.25% | 0.00 | 0.00 | 0 |
| 349 | 41.00% | 0.00 | 0.00 | 0 |
| 350 | 40.86% | 0.50 | 0.50 | 0 |
| 351 | 37.50% | 0.00 | 0.00 | 0 |
| 352 | 39.00% | 0.00 | 0.00 | 0 |
| 353 | 40.00% | 0.00 | 0.00 | 0 |
| 354 | 34.25% | 0.00 | 0.00 | 0 |
| 355 | 40.00% | 0.00 | 0.00 | 0 |
| 356 | 38.00% | 0.00 | 0.00 | 0 |
| 357 | 36.25% | 0.00 | 0.00 | 0 |
| 358 | 35.00% | 0.00 | 0.00 | 0 |
| 359 | 37.95% | 0.00 | 0.67 | 0 |
| 360 | 35.06% | 0.50 | 0.00 | 0 |
| 361 | 39.50% | 0.00 | 0.00 | 0 |
| 362 | 40.10% | 0.00 | 0.67 | 0 |
| 363 | 39.25% | 0.00 | 0.00 | 0 |
| 364 | 41.50% | 0.00 | 0.00 | 0 |
| 365 | 36.75% | 0.00 | 0.00 | 0 |
| 366 | 37.00% | 0.00 | 0.00 | 0 |
| 367 | 41.91% | 0.00 | 0.50 | 0 |
| 368 | 41.25% | 0.00 | 0.00 | 0 |
| 369 | 41.19% | 0.00 | 1.00 | 0 |
| 370 | 46.25% | 0.00 | 0.00 | 0 |
| 371 | 39.50% | 0.00 | 0.00 | 1 |
| 372 | 34.61% | 5.00 | 0.50 | 0 |
