# Supplementary material for: A comparative plastomics approach reveals available molecular markers for the phylogeographic study of Dendrobium huoshanense, an endangered orchid with extremely small populations
Source: Ecol Evol. 2020 Apr 30;10(12):5332–42. doi: 10.1002/ece3.6277 (PMC7319108; doi:10.1002/ece3.6277)
Supplement: Supplementary file 7 — Table S3 [file ECE3-10-5332-s007.docx]

| Table S3. The 27 mutational hotspot regions. | |
| --- | --- |
| Bins | Location |
| 9 | *trnK-intron2* |
| 10 | *trnK-intron2* |
| 12 | *trnK-rps16* |
| 16 | *rps16-trnQ* |
| 17 | *rps16-trnQ* |
| 19 | *psbK-trnS* |
| 20 | *psbK-trnS* |
| 22 | *trnS-trnG* |
| 24 | *trnG-intron* |
| 69 | *rpoB-trnC* |
| 70 | *rpoB-trnC* |
| 71 | *rpoB-trnC* |
| 72 | *rpoB-trnC* |
| 73 | *trnC-petN* |
| 74 | *trnC-petN* |
| 124 | *trnF-trnV* |
| 125 | *trnF-trnV* |
| 126 | *trnF-trnV* |
| 127 | *trnF-trnV* |
| 128 | *trnF-trnV* |
| 180 | *clpP-intron2* |
| 181 | *clpP-intron2* |
| 182 | *clpP-intron2* |
| 252 | *rps12-trnV* |
| 253 | *rps12-trnV* |
| 254 | *rps12-trnV* |
| 255 | *rps12-trnV* |
