# Supplementary material for: A comparative plastomics approach reveals available molecular markers for the phylogeographic study of Dendrobium huoshanense, an endangered orchid with extremely small populations
Source: Ecol Evol. 2020 Apr 30;10(12):5332–42. doi: 10.1002/ece3.6277 (PMC7319108; doi:10.1002/ece3.6277)
Supplement: Supplementary file 8 — Table S4 [file ECE3-10-5332-s008.docx]

| Table S4. Location of the six polymorphic cpSSRs. | | |
| --- | --- | --- |
| Bins | Repeat motif | Location |
| 9 | (AT)_9_ | *trnK-intron2* |
| 17 | A_12_ | *rps16-trnQ* |
| 20 | A_8_ | *psbK-trnS* |
| 72 | T_7_ | *rpoB-trnC* |
| 74 | (TA)_5_ | *trnC-petN* |
| 118 | A_10_ | *rps4-trnT* |
