# Supplementary material for: A comparative plastomics approach reveals available molecular markers for the phylogeographic study of Dendrobium huoshanense, an endangered orchid with extremely small populations
Source: Ecol Evol. 2020 Apr 30;10(12):5332–42. doi: 10.1002/ece3.6277 (PMC7319108; doi:10.1002/ece3.6277)
Supplement: Supplementary file 9 — Table S5 [file ECE3-10-5332-s009.docx]

| Table S5. Sequence polymorphisms of cpDNA haplotype. | | | | | | | | | | | |
| --- | --- | --- | --- | --- | --- | --- | --- | --- | --- | --- | --- |
| Position | Hap 1 | Hap 2 | Hap 3 | Hap 4 | Hap 5 | Hap 6 | Hap 7 | Hap 8 | Hap 9 | Hap 10 | Hap 11 |
| 858 | T | . | G | . | . | . | . | . | . | . | . |
| 3109 | 0 | 1 | 1 | 1 | 1 | 1 | . | . | 1 | 1 | 1 |
| 3781 | A | . | . | . | . | T | . | . | . | . | . |
| 4541 | T | . | G | . | . | . | . | . | . | . | . |
| 4830 | A | . | . | . | . | . | . | . | G | G | G |
| 5042 | - | - | - | - | - | - | - | - | - | T | - |
| 5311 | G | . | . | . | C | . | . | . | . | . | . |
| 5505 | T | . | - | . | . | . | . | . | . | . | . |
| 5534 | G | . | A | . | . | . | . | . | . | . | . |
| 5753 | 0 | - | - | - | - | 2 | 2 | - | - | - | - |
| 8384 | T | . | . | . | . | . | . | G | . | . | . |
| 9688 | 3 | . | . | . | . | . | . | . | 0 | 0 | . |
| 10051 | A | C | . | . | C | C | . | . | C | C | C |
| 10053 | T | A | . | . | A | A | . | . | A | A | A |
| Note: All sequences are compared to that of the reference haplotype H1. Note that all mononucleotide repeats were excluded from the analysis. “.”: indicate the same nucleotide with haplotype H1. “-”: denotes a single nucleotide InDel. Numbers ‘0/1/2/3’ in the sequences indicate absence or presence of length polymorphisms: 0, absence of the InDel; 1, CTTTTTGCTATTCACCTATTCTTTTTTTTTTTTCAATCCCGCGCCGCAGCAGAACAAAA; 2, ATTCTATTT; 3, TCTATCC. | | | | | | | | | | | |
